# High-speed vibration-milling-promoted synthesis of symmetrical frameworks containing two or three pyrrole units

**DOI:** 10.3762/bjoc.13.190

**Published:** 2017-09-15

**Authors:** Marco Leonardi, Mercedes Villacampa, J Carlos Menéndez

**Affiliations:** 1 Departamento de Química Orgánica y Farmacéutica, Facultad de Farmacia, Universidad Complutense, 28040 Madrid, Spain

**Keywords:** diversity-oriented synthesis, mechanochemistry, multicomponent reactions, pyrroles, solvent-free synthesis

## Abstract

The pseudo-five-component reaction between β-dicarbonyl compounds (2 molecules), diamines and α-iodoketones (2 molecules), prepared in situ from aryl ketones, was performed efficiently under mechanochemical conditions involving high-speed vibration milling with a single zirconium oxide ball. This reaction afforded symmetrical frameworks containing two pyrrole or fused pyrrole units joined by a spacer, which are of interest in the exploration of chemical space for drug discovery purposes. The method was also extended to the synthesis of one compound containing three identical pyrrole fragments via a pseudo-seven-component reaction. Access to compounds having a double bond in their spacer chain was achieved by a different approach involving the homodimerization of 1-allyl- or 1-homoallylpyrroles by application of cross-metathesis chemistry.

## Introduction

Symmetrical molecules formed by two or more pharmacophoric units joined by a spacer are very important in drug discovery because many drug targets are symmetrical, the reason most often being that they are composed by two or more identical subunits. Some examples of therapeutic targets in which symmetrical bivalent ligands have proved to be useful include the protease of the human immunodeficiency virus HIV [1], cellular prion protein, PrP^c^ [2], and the transient receptor potential melastatin 8 (TRPM8) channel receptor [3]. The synthesis of these symmetrical molecules normally relies on multistep sequences. Due to the special relevance of nitrogen heterocycles in the generation of bioactive molecules, medicinal chemists would greatly benefit from the ability to build two heterocyclic systems at both ends of the spacer chain in a single maneuver. Further advantages in terms of synthetic efficiency would be gained if the key operations leading to the buildup of the heterocyclic frameworks could be performed using multicomponent reactions. However, the simultaneous construction of two heterocycles in a single operation by means of such reactions has very little precedent in the literature and has never been achieved under mechanochemical conditions.

**Scheme 1:**
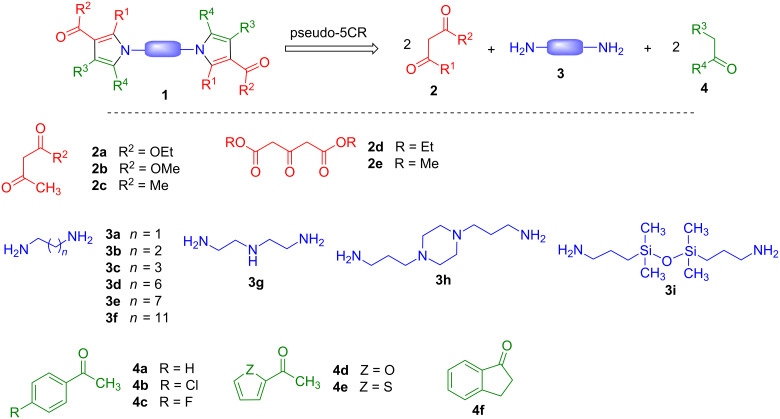
Our synthetic planning and structural diversity of starting materials employed in our work.

In this context, we present here our results on the development of pseudo-five-component reactions allowing the construction of bispyrrolic systems **1** starting from β-dicarbonyl compounds **2**, diamine derivatives **3** and aryl ketones **4**, together with some related additional methodology. The disconnection employed and the structural diversity introduced at the three reaction components is summarized in Scheme 1.

Our procedure involves the use of mechanochemistry, which deals with reactions promoted by mechanical energy and is emerging in recent years as a versatile tool that allows solvent-free approaches to organic and inorganic synthesis [4–13]. Indeed, the protocol reported here can be viewed as a generalization of our previously published pyrrole synthesis based on the reaction between primary amines, β-dicarbonyl compounds and ketones, promoted by high-speed vibration milling (HSVM) [14–15]. The importance of pyrrole frameworks stems from the status of this heterocycle as a privileged structure in drug discovery due to its presence as a structural core in molecules that are able to bind various receptors [16].

## Results and Discussion

**Scheme 2:**
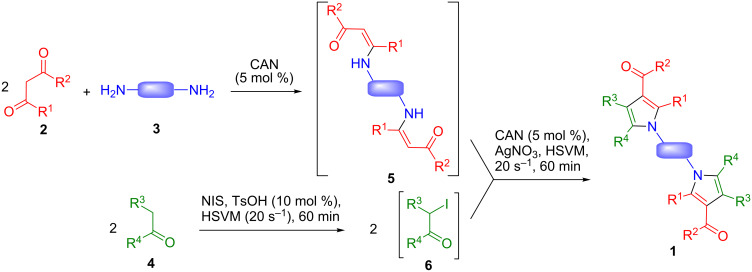
Pseudo five-component reactions affording symmetrical bispyrrole derivatives joined by a spacer.

Our route to the target bispyrrole systems is summarized in Scheme 2. Treatment of aromatic ketones **4** with *N*-iodosuccinimide (NIS) in the presence of toluenesulfonic acid under high-speed vibration milling for 1 h afforded α-iodoketones **6**, which were not isolated. The suitable β-dicarbonyl compound **2** and α,ω-diamine **3** plus a catalytic amount of Ce(IV) ammonium nitrate (CAN) [17], which had been previously pre-mixed for 30–60 min to ensure the complete generation of the intermediate bis-β-enaminones **5**, were added to the reaction vessel, together with silver nitrate, and the mixture was again submitted to milling for an additional hour. The reactions were routinely performed from 1 mmol of the starting materials, but two of them (leading to compounds **1a** and **1n**) were also carried out at a 10 mmol scale without any significant loss in yield.

This solvent-free protocol combines the initial α-iodination of the starting ketone **4** with a three-component pyrrole synthesis related to the classical Hantzsch reaction. The intermediacy of species **5** and **6** was proved by the following experimental observations:

1. They could be isolated by suitably interrupting our process. As a representative example, the bis-β-enaminone arising from methyl acetoacetate and 1,4-butanediamine was isolated in quantitative yield after mixing the starting materials in the presence of 5% CAN. The isolation and characterization of the intermediate α–iodoketones has been reported previously [15].
2. The isolated intermediates reacted under our usual conditions to give pyrrole derivatives **1**.

In some cases (compounds **1d**, **1g**, **1j**, **1l**, **1m**, **1o**), the ball milling-promoted iodination step failed and it was necessary to obtain the α-iodoketones **6** in a separate step by treatment of **4** with I_2_ and CuO in methanol. The iodination of 1-indanone (eventually leading to **1o**) may be hampered by steric hindrance, since this is the only case where R^3^ is different from H. In the cases of 2-furyl methyl ketone and 2-thienyl methyl ketone, the reason for the lower reactivity under solvent-free conditions may be the stabilization of the intermediate enol via intramolecular hydrogen bonding, which would be disrupted in the alternative conditions involving the use of methanol as solvent.

**Figure 1:**
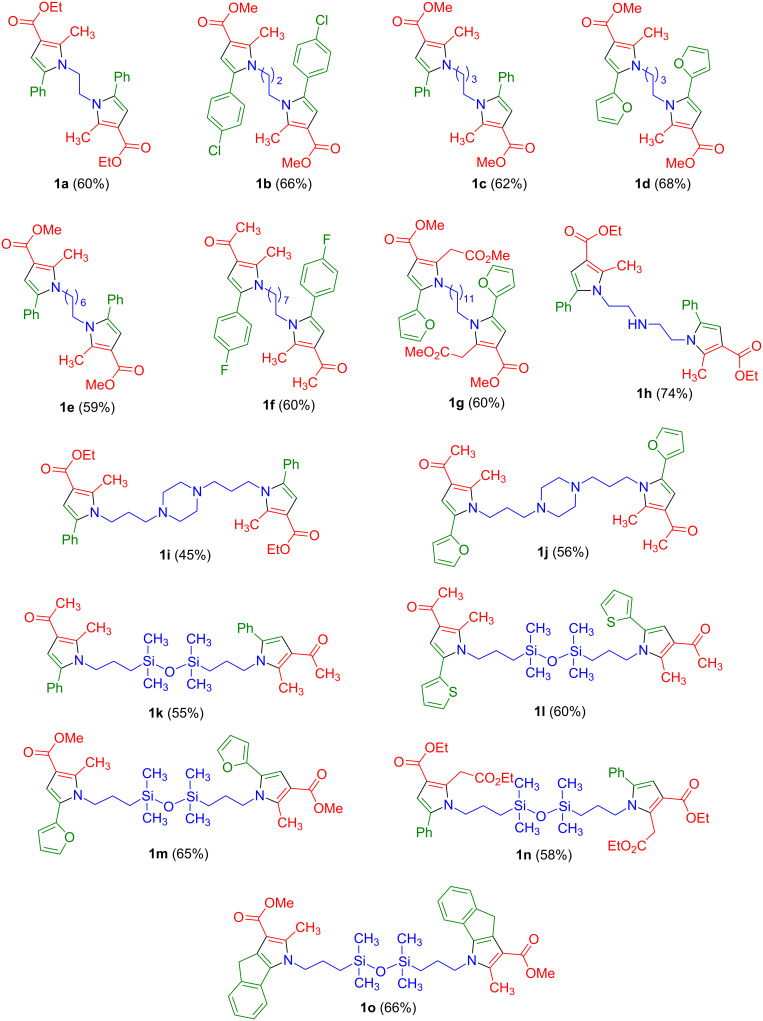
Scope of the synthesis of symmetrical bispyrrole derivatives.

The scope of the method is summarized in Figure 1. In some cases (compounds **1a**–**g**) the spacer was simply a polymethylene chain, but the inclusion of spacer chains containing an amino group (**1h**), piperazine (**1i**,**j**) or tetramethyldisiloxane (**1k**–**o**) fragments, was also feasible. Interestingly, the use of *N*^1^-(2-aminoethyl)ethane-1,2-diamine as the starting material was also possible, without interference from the secondary amino group in spite of its nucleophilicity, to give compound **1h**. This kind of functionalized spacers is interesting in that they may allow additional interactions with biological targets. Furthermore, the tetramethyldisiloxane derivatives are of relevance in view of the current interest in silicon-containing compounds for drug discovery applications, which has led to the “silicon switch” approach to the design of bioactive molecules [18–20]. Regarding the pyrrole rings, they were generally methyl-substituted at C-2, but the attachment of functional groups to the methyl substituent was also possible, as shown by the preparation of compounds **1g** and **1n**. Ketone (compounds **1f** and **1j**–**l**) and ester functions (compounds **1a**–**e**, **1g**–**i** and **1m**–**o**) could be present at C-3, although an attempt to introduce an amide was unsuccessful. A variety of aromatic and heteroaromatic rings could be present at the C-5 position, and the synthesis of compound **1o** from 1-indanone proved the possibility to prepare systems containing two linked fused pyrrole moieties.

The advantages of the mechanochemical Hantzsch protocol over the conventional one in solution in terms of yield, reaction time and, in most cases, the possibility to telescope the formation of the α-iodide and the pyrrole synthesis in a single process have been previously established [15]. Nevertheless, in order to achieve a more reliable extension of this conclusion to the pseudo-5CR reactions described in the present article, we have performed a control experiment with the reaction leading to **1c** and observed a significantly lower yield (47% vs 62%) and a longer reaction time (5 h vs 2 h) in solution.

**Scheme 3:**
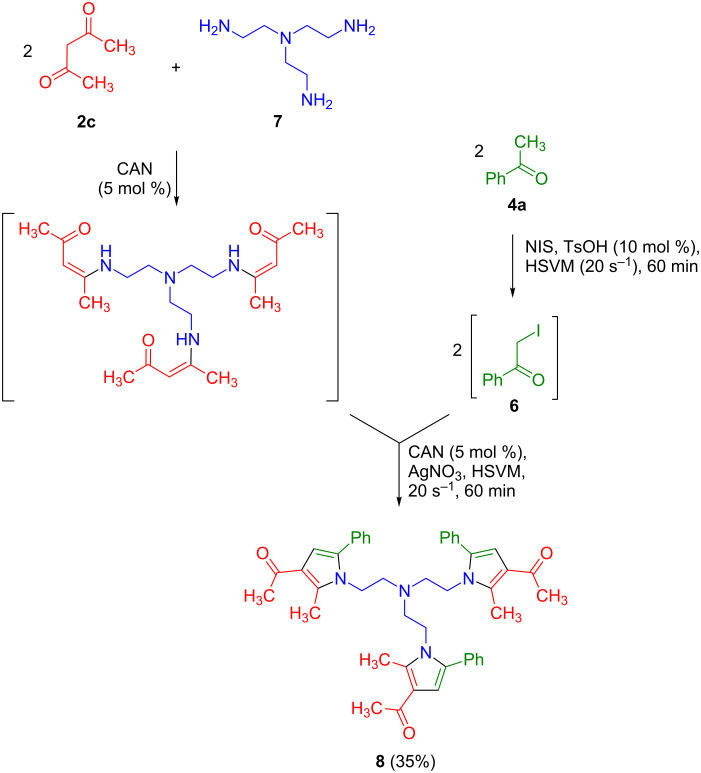
A pseudo-seven-component reaction that affords a terpyrrole derivative with a functionalized spacer.

The use of triamine **7** as the starting material allowed the preparation of compound **8** via a pseudo-seven-component reaction (Scheme 3). While the overall yield was only moderate, it has to be taken into account that the preparation of **8** involves 12 individual steps, with a linear sequence comprising 9, and thus the average yield is 89% per step. In view of its functionalization, compound **7** can be regarded as a good precursor to heterocyclic dendrimeric structures.

**Scheme 4:**
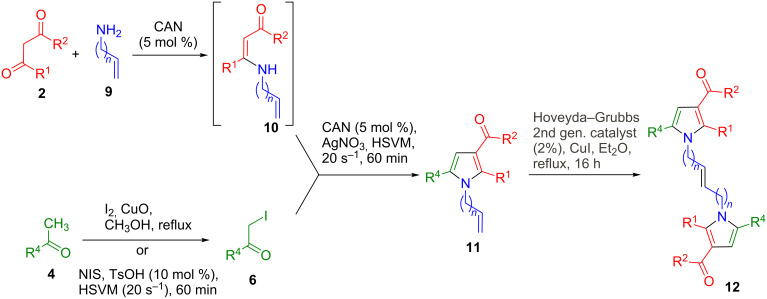
Homodimerization of 2-allyl- and 2-homoallylpyrroles via cross-metathesis reactions.

As an additional entry into symmetrical systems containing two pyrrole structural fragments, we briefly examined the homodimerization reactions of 2-allyl- and 2-homoallylpyrroles via cross-metathesis, which should give access to spacers not easily accessible by the previously described route. The starting materials for this study (compounds **11**) were readily prepared under the conditions for single-ring pyrrole derivatives [14–15] and, as shown in Scheme 4, they were uneventfully transformed into the target compounds **12** in the presence of the second-generation Hoveyda–Grubbs catalyst and copper(I) iodide. Interestingly, the reactions starting from 1-allylpyrroles gave a single stereoisomer at the central double bond, which was assumed to be *E*, while compound **12c**, obtained from a 1-homoallyl derivative, was isolated as a 1:1 *E*/*Z* mixture (Table 1).

**Table 1:** Yields of the cross-metathesis reactions.

Cmpd	R^1^	R^2^	R^4^	*n*	**11**, %	**12**, %

**a**	CH_3_	OMe	2-furyl	1	87	88
**b**	CH_3_	Me	C(CH_3_)_3_	1	60	70^a^
**c**	CH_3_	OMe	2-thienyl	2	71	80^b^
**d**	CH_2_-CO_2_Et	OEt	2-furyl	1	80	88

^a^The reaction time was 48 h in this case. ^b^As a 1:1 *E*/*Z* mixture.

## Conclusion

Symmetrical compounds containing two or three pyrrole or fused pyrrole units joined by a spacer are of interest in the exploration of heterocyclic chemical space. Such compounds were readily accessible in a single operation via the construction of their pyrrole fragments by means of mechanochemical multicomponent reactions that were performed starting from very simple starting materials and catalysts. Related compounds having a double bond in their spacer chain were obtained by a different approach involving the homodimerization of 1-allyl- or 1-homoallylpyrroles by cross-metathesis.

## Supporting Information

File 1 Experimental details and NMR spectra.
