# Supplementary material for: High-speed vibration-milling-promoted synthesis of symmetrical frameworks containing two or three pyrrole units
Source: Beilstein J Org Chem. 2017 Sep 15;13:1957–62. doi: 10.3762/bjoc.13.190 (PMC5629381; doi:10.3762/bjoc.13.190)

Supporting information

for

High-speed vibration-milling-promoted synthesis of symmetrical frameworks containing two or three pyrrole units

Marco Leonardi, Mercedes Villacampa and J. Carlos Menéndez*

Address: Departamento de Química Orgánica y Farmacéutica, Facultad de Farmacia, Universidad Complutense, 28040 Madrid, Spain

Email: J. Carlos Menéndez - josecm@ucm.es

*Corresponding author

Experimental details and NMR spectra

Table of contents

1.- General experimental details S2

2.- General procedure for the synthesis of symmetrical bispyrrole derivatives **1** under solvent-free high-speed vibration milling (HSVM) conditions S2

3.- General procedure for the synthesis of pyrroles derivatives **11** under solvent-free high-speed vibration milling (HSVM) conditions S9

4.- Preparation of compounds **12** by cross-metathesis reactions S11

5.- Copies of ^{1}H, ^{13}C and ^{19}F NMR spectra S13

1. General experimental details

All reagents (Aldrich, Fischer, Alpha Aesar) and solvents (Scharlau, Fischer) were of commercial quality and were used as received. Mechanochemical reactions were carried out in a Retsch MM200 mixer mill at a frequency of 20 Hz using a 25 mL zirconium oxide grinding jar and a single zirconium oxide ball 20 mm in diameter. Reactions were monitored by thin layer chromatography on aluminium plates coated with silica gel and fluorescent indicator (Macherey-Nagel Xtra SIL G/UV_{254}). Separations by flash chromatography were performed on silica gel (Scharlau 40–60 μm, 230–400 mesh ASTM). Melting points were determined using a Stuart Scientific apparatus, SMP3 Model, and are uncorrected. Infrared spectra were recorded with an Agilent Cary630 FTIR spectrophotometer with a diamond accessory for solid and liquid samples. NMR spectroscopic data were recorded using a Bruker Avance 250 spectrometer operating at 250 MHz for ^{1}H NMR and 63 MHz for ^{13}C NMR (CAI de Resonancia Magnética Nuclear, Universidad Complutense); chemical shifts are given in ppm and coupling constants in Hertz. High-resolution mass spectra (HRMS) were recorded on a mass spectrometer fitted with an electrospray detector (ESI) by the CAI de Espectrometría de Masas, Universidad Complutense. Elemental analyses were determined by the CAI de Microanálisis Elemental, Universidad Complutense, using a Leco 932 combustion microanalyzer.

2. General procedure for the synthesis of symmetrical bispyrrole derivatives 1 under solvent-free high-speed vibration milling (HSVM) conditions

The suitable ketone (1 mmol), *N*-iodosuccinimide (NIS, 1 mmol) and *p*-toluenesulphonic acid (PTSA, 10 mol %) were added to a commercially available snap closure grinding jar, along with a zirconium oxide ball. This ball mill vessel was fitted to one of the horizontal vibratory arms of the ball mill, while the other arm was occupied with an empty vessel. The ball mill was set to vibrate at a frequency of 20 s^{-1} for 60 min at room temperature. Then, a mixture of the corresponding diamine (0.85 mmol), the suitable β-dicarbonyl compound (1.3 mmol) and cerium(IV) ammonium nitrate (CAN, 0.13 mmol) in 0.5 mL of methanol was stirred at room temperature during 30–60 min (judged by TLC), and the solvent was evaporated. The residue was transferred to the milling vessel with a Pasteur pipette or a spatula and silver nitrate (1 mmol) was added. The reaction was subjected to the vibratory movement at 20 s^{-1} for 60 min, affording a dark paste. Then, the reaction vessel was cleansed with ethyl acetate or dichloromethane and the suspension was filtered to remove the silver iodide precipitate. The organic layer was washed with water (2 mL), dried over anhydrous sodium sulfate and the solvent was removed under reduced pressure. Purification by flash column chromatography on silica gel eluting with a gradient from petroleum ether to 8:2 petroleum ether–ethyl acetate afforded the desired pyrrole derivatives. Compounds **1h**, **1i** and **1j** were purified by flash chromatography eluting with a 98/2 dichloromethane/methanol mixture.

For the synthesis of compounds **1d, 1g, 1j, 1l, 1m, 1o**, α-iodoketones were prepared in a separate step, according to the procedure described below. These iodides were known in the literature.[1]

To a solution of the suitable ketone (1 equiv) in anhydrous methanol, iodine (1 equiv) and cupper(II) oxide (1 equiv) were added. The mixture was stirred at room temperature for 5 min and, then, refluxed until no starting material was detected by TLC. The reaction was cooled, filtered and the solvent was removed. The residue was dissolved in ethyl acetate (10 mL) and washed with a 10% solution of $Na_2S_2O_3$ (20 mL). The aqueous phase was extracted with ethyl acetate (2 × 20 mL) and the combined organic layers were dried over anhydrous sodium sulfate and the solvent was evaporated.[2] The α-iodoketones thus obtained were used in the next reaction without further purification.

Diethyl 1,1'-(ethane-1,2-diyl)bis(2-methyl-5-phenyl-1*H*-pyrrole-3-carboxylate) (1a)

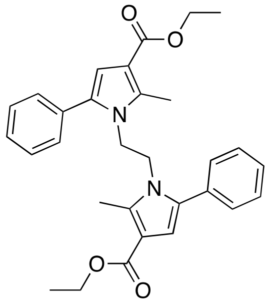

Prepared from acetophenone (1 mmol), ethylenediamine (0.85 mmol) and ethyl acetoacetate (1.30 mmol); yield: 145 mg (60%); white solid; mp: 224-226 °C; ^{1}H NMR ($CDCl_3$, 250 MHz) δ 7.42-7.36 (m, 6H); 7.20-7.15 (m, 4H); 6.48 (s, 2H); 4.28 (q, *J* = 7.1 Hz, 4H); 3.88 (s, 4H); 1.98 (s, 6H); 1.36 (t, *J* = 7.1 Hz, 6H) ppm; ^{13}C NMR ($CDCl_3$, 63 MHz) δ 165, 136.1, 133.0, 132.0, 129.7, 128.6, 128.0, 112.4, 110.3, 59.3, 43.1, 14.5, 10.2 ppm; IR (neat) ν: 1701.6 (C=O), 1242.9 (C-O) cm^{-1}. Elemental analysis (%) calcd for $C_{30}H_{32}N_2O_4$: C 74.36, H 6.66, N 5.78; found: C 74.31, H 6.59, N 5.80.

Dimethyl 1,1'-(propane-1,3-diyl)bis[5-(4-chlorophenyl)-2-methyl-1*H*-pyrrole-3-carboxylate] (1b)

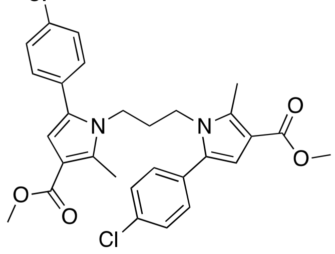

Prepared from 4-chloro-acetophenone (1 mmol), propane-1,3-diamine (0.85 mmol) and methyl acetoacetate (1.3 mmol); yield: 178 mg (66%); white solid; mp: 84-86; ^{1}H NMR (250 MHz, $CDCl_3$) δ 7.37 – 7.26 (m, 4H), 7.13 – 7.05 (m, 4H), 6.48 (d, *J* = 0.9 Hz, 2H), 3.81 (s, 6H), 3.71 – 3.66 (m, 4H), 2.44 (s, 6H), 1.69 – 1.60 (m, 2H); ^{13}C NMR (63 MHz, $CDCl_3$) δ 165.4, 136.2, 133.6, 131.6, 130.7, 130.0, 128.7, 112.1, 110.4, 50.7, 40.7, 31.2, 11.2; IR (neat) ν: 2946.4 (C-H), 1689.4 (C=O),

1 Estévez, V.; Sridharan, V.; Sabaté, S.; Villacampa, M.; Menéndez, J. C. *Asian J. Org. Chem*. **2016**, *5*, 652–662.

2 Yin, G.; Gao, M.; She, N.; Hu, S.; Wu, A.; Pan, Y. *Synthesis* **2007**, 3113–3116.

1242.7 (C-O), 1010.9 (C-Cl) cm^{-1}; elemental analysis (%) calcd. for $C_{29}H_{28}Cl_2N_2O_4$: C, 64.57; H, 5.23; N, 5.19; found: C, 64.53; H, 5.20; N, 5.14.

Dimethyl 1,1'-(butane-1,4-diyl)bis(2-methyl-5-phenyl-1*H*-pyrrole-3-carboxylate) (1c)

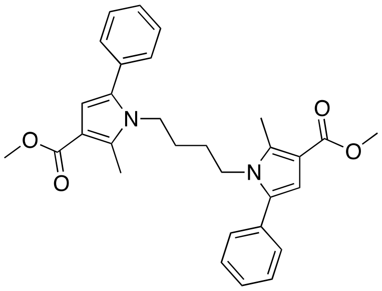

Prepared from acetophenone (1 mmol), butane-1,4-diamine (0.85 mmol) and methyl acetoacetate (1.3mmol); yield: 150 mg (62%); yellowish solid; mp: 156-158 °C; ^{1}H NMR (250 MHz, $CDCl_3$) δ 7.15 (m, 6H), 7.02 – 6.93 (m, 4H), 6.27 (s, 2H), 3.59 (s, 6H), 3.46 (m, 4H), 2.27 (s, 6H), 1.01 (m, 4H); ^{13}C NMR (63 MHz, $CDCl_3$) δ 165.9, 136.2, 133.2, 132.8, 129.2, 128.4, 127.6, 111.6, 109.8, 50.7, 43.0, 27.1, 11.4; IR (neat) ν: 2953.2, 2877.8 (C-H), 1690.5 (C=O), 1243.2, 1211.6 (C-O) cm^{-1}; HRMS (ESI) *m/z* calcd. for $[M+H]^+$ 485.24348, found 485.24568; elemental analysis (%) calcd. for $C_{30}H_{32}N_2O_4$: C, 74.36; H, 6.66; N, 5.78; found: C, 74.31; H, 6.63; N, 5.77.

Dimethyl 1,1'-(butan-1,3-diyl)bis[5-(furan-2-yl)-2-methyl-1*H*-pyrrole-3-carboxylate] (1d)

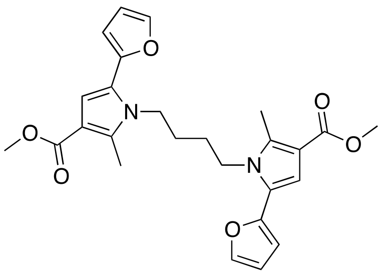

Prepared from 2-furyl methyl ketone (1 mmol), butane-1,4-diamine (0.85 mmol) and methyl acetoacetate (1.3 mmol); yield: 158 mg (68%); yellowish oil; ^{1}H NMR (250 MHz, $CDCl_3$) δ 7.43 (dd, *J* = 1.9, 0.8 Hz, 2H), 6.74 (s, 2H), 6.47 (dd, *J* = 3.3, 1.9 Hz, 2H), 6.36 (dd, *J* = 3.3, 0.8 Hz, 2H), 3.97 (m, 4H), 3.83 (s, 6H), 2.56 (s, 6H), 1.82 – 1.58 (m, 4H); ^{13}C NMR (63 MHz, $CDCl_3$) δ 165.7, 146.8, 141.7, 136.9, 123.1, 111.9, 111.2, 110.6, 107.5, 50.9, 44.3, 27.5, 11.2; IR (neat) ν: 2945.0 (C-H), 1700.5 (C=O), 1249.1(C-O) cm^{-1}; elemental analysis (%) calcd. for $C_{26}H_{28}N_2O_6$: C, 67.23; H, 6.08; N, 6.03; found: C, 67.13; H, 6.01; N, 5.99.

Dimethyl 1,1'-(heptane-1,7-diyl)bis(2-methyl-5-phenyl-1*H*-pyrrole-3-carboxylate) (1e)

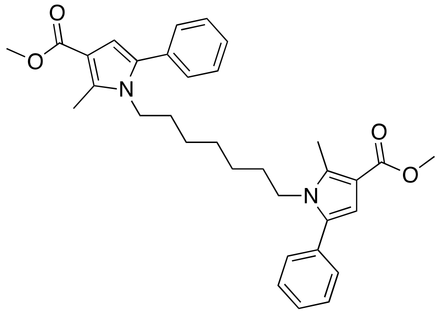

Prepared from acetophenone (1 mmol), heptane-1,7-diamine (0.85 mmol) and methyl acetoacetate (1.3 mmol); yield: 155 mg (59%); yellow solid; mp: 96-98 °C; ^{1}H NMR (250 MHz, $CDCl_3$) δ 7.43 – 7.29 (m, 10H), 6.54 (s, 2H), 3.92 – 3.81 (m, 4H), 3.87 (s, 6H), 2.61 (s, 6H), 1.47 –1.41 (m, 4H), 0.99 (s, 6H); ^{13}C NMR (63 MHz, $CDCl_3$) δ 166.0, 136.4, 133.4, 133.0, 129.3, 128.4, 127.4, 111.5, 109.6, 50.7, 43.8,

30.3, 28.2, 26.1, 11.5; IR (neat) ν: 2935.9, 2900.0 (C-H), 1729.0 (C=O), 1245.3 (C-O) cm^{-1}; elemental analysis (%) calcd. for $C_{33}H_{38}N_2O_4$: C, 75.26; H, 7.27; N, 5.32; found: C, 75.26; H, 7.28; N, 5.30.

1,1'-(Octane-1,8-diyl)bis[5-(4-fluorophenyl)-2-methyl-1*H*-pyrrole-1,3-diyl]bis(ethan-1-one) (1f)

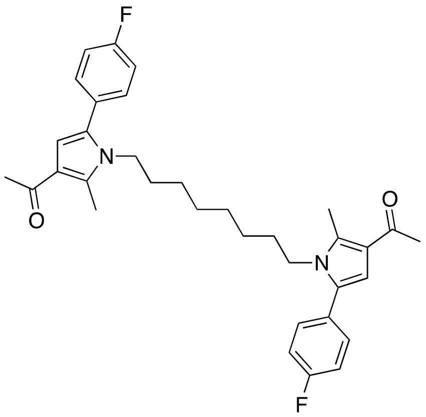

Prepared from 1-(4-fluorophenyl)ethan-1-one (1 mmol), octane-1,8-diamine (0.85 mmol) and acetylacetone (1.3mmol); yield: 163 mg (60%); yellowish solid; mp: 158-160 °C; ^{1}H NMR (250 MHz, $CDCl_3$) δ 7.24 – 7.09 (m, 4H), 7.02 – 6.88 (m, 4H), 6.29 (s, 2H), 3.79 – 3.54 (m, 4H), 2.45 (s, 6H), 2.26 (s, 6H), 1.29 – 1.25(m, 4H), 0.96 – 0.81 (m, 8H); ^{13}C NMR (63 MHz, $CDCl_3$) δ 195.0, 162.3 (d, *J* = 252 Hz), 135.7, 131.9, 131.2 (d, *J* = 7.6 Hz), 129.0 (d, *J* = 3.8 Hz), 120.8, 115.4 (d, *J* = 21.4 Hz), 110.5, 43.6, 30.4, 28.7, 28.5, 26.3, 12.0; ^{19}F NMR (235 MHz, $CDCl_3$) δ (-114.31) – (-114.43) (m); IR (neat) ν: 2932.7, 2851.1 (C-H), 1649.2 (C=O), 1242.9, 1215.5 (C-O), 1152.1 (C-F) cm^{-1}; elemental analysis (%) calcd. for $C_{34}H_{38}F_2N_2O_2$: C, 74.97; H, 7.03; N, 5.14; found: C, 74.91; H, 7.00; N, 5.10.

Dimethyl 1,1'-(dodecane-1,12-diyl)bis[5-(furan-2-yl)-2-(2-methoxy-2-oxoethyl)-1*H*-pyrrole-3-carboxylate] (1g)

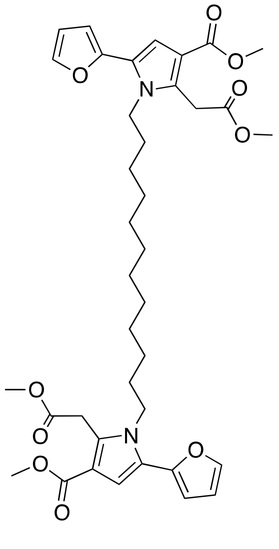

Prepared from 2-furyl methyl ketone (1 mmol), dodecane-1.12-diamine (0.85 mmol) and dimethyl 3-oxopentanedioate (1.3 mmol); yield: 207 mg (60%) ; transparent oil; ^{1}H NMR (250 MHz, $CDCl_3$) δ 7.48 – 7.46 (m, 2H), 6.80 (s, 2H), 6.49 – 6.47 (m, 2H), 6.42 – 6.41 (m, 2H), 4.16 (s, 4H), 4.06 – 3.96 (m, 4H), 3.82 (s, 6H), 3.74 (s, 6H), 1.71 – 1.56 (m, 4H), 1.35 – 1.17 (m, 16H); ^{13}C NMR (63 MHz, $CDCl_3$) δ 170.3, 165.3, 146.6, 141.8, 132.3, 124.3, 113.2, 111.1, 110.6, 107.6, 52.3, 51.0, 45.4, 31.1, 30.7, 29.5, 29.4, 29.1, 26.6; IR (neat) ν: 2926.0, 2852.6 (C-H), 1740.8, 1703.4 (C=O), 1250.1, 1233.2 (C-O) cm^{-1}; elemental analysis (%) calcd. for $C_{38}H_{48}N_2O_{10}$: C, 65.88; H, 6.98; N, 4.04; found: C, 65.89; H, 6.92; N, 4.04.

N,*N*-Bis[2-(3-ethoxycarbonyl-5-phenyl-2-methyl-1*H*-pyrrol-1-yl)ethyl]amine (1h)

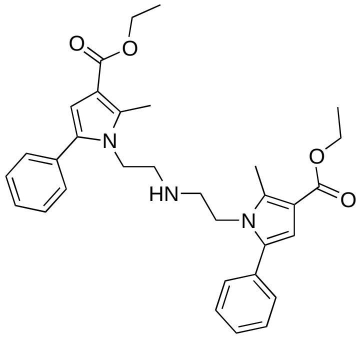

Prepared from acetophenone (1 mmol), *N*-(2-aminoethyl)ethane-1,2-diamine (0.85 mmol) and ethyl acetoacetate (1.3 mmol); yield: 183 mg (74%); yellowish oil; ^{1}H NMR ($CDCl_3$, 250 MHz) δ 7.15-7.00 (m, 10H), 6.36 (s, 2H), 4.12 (q, *J* = 7.1 Hz, 4H), 3.69 (t, *J* = 6.8 Hz, 4H), 2.37 (s, 6H), 2.26 (t, *J* = 6.8 Hz, 4H), 1.18 (t, *J* = 7.1 Hz, 6H) ppm; ^{13}C NMR ($CDCl_3$, 63 MHz) δ 165.5, 136.5, 133.3, 132.8, 129.3, 128.5, 127.6, 112.1, 110.1, 59.3, 48.8, 43.7, 14.5, 11.6 ppm; IR (neat) ν: 1650 (C=O), 1250 (C-O) cm^{-1}; Elemental analysis (%) calcd for $C_{32}H_{37}N_3O_4$: C 72.84, H 7.07, N 7.96; C 72.79, H 7.03, N 7.91.

*N*1,*N*4-Bis[3-(3-ethoxycarbonyl-5-phenyl-2-methyl-1*H*-pyrrol-1-yl)propyl]piperazine (1i)

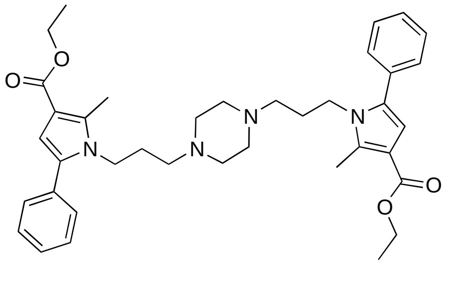

Prepared from acetophenone (1 mmol), 1,4-bis(aminopropyl)piperazine (0.65 mmol) and ethyl acetoacetate (1.3 mmol); yield: 141 mg (45%); yellowish oil; ^{1}H NMR ($CDCl_3$, 250 MHz) δ 7.21-7.06 (m, 10H), 6.34 (s, 2H), 4.08 (q, *J* = 7.1 Hz, 4H), 3.73 (m, 4H), 2.41 (s, 6H), 1.97-1.85 (m, 12H), 1.45-1.34 (m, 4H), 1.14 (t, *J* = 7.1, 6H) ppm; ^{13}C NMR ($CDCl_3$, 63 MHz) δ 166.0, 136.9, 133.6, 133.5, 129.7, 128.8, 127.8, 112.3, 110.3, 59.7, 55.2, 53.1, 42.5, 27.9, 14.9, 11.9; IR (neat) ν: 1650 (C=O), 1250 (C-O) cm^{-1}; elemental analysis (%) calcd for $C_{38}H_{48}N_4O_4$: C 73.05, H 7.74 N, 8.97; found: C 72.97, H 7.70, N 8.90.

*N*1,*N*4-Bis[3-[3-(methylcarbonyl)-5-(furan-2-yl)-2-methyl-1*H*-pyrrol-1-yl]propyl]piperazine (1j)

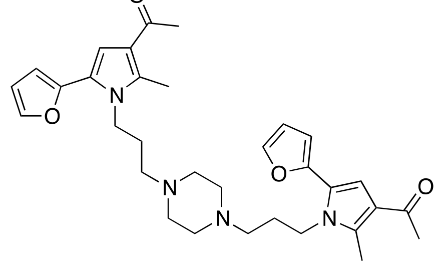

Prepared from 2-furyl methyl ketone (1 mmol), 3,3'-(piperazine-1,4-diyl)bis(propan-1-amine) (0.85 mmol) and acetylacetone (1.3 mmol); yield: 152 mg (56%); light brown oil; ^{1}H NMR (250 MHz, $CDCl_3$) δ 7.37 (dd, *J* = 1.9, 0.8 Hz, 2H), 6.63 (s, 2H), 6.39 (dd, *J* = 3.3, 1.9 Hz, 2H), 6.35 (dd, *J* = 3.3, 0.8 Hz, 2H), 4.02 – 3.95 (m, 4H), 2.54 (s, 6H), 2.41 – 2.16 (m, 12H), 2.34 (s, 6H), 1.81 – 1.69 (m, 4H); ^{13}C NMR (63 MHz, $CDCl_3$) δ 194.9, 146.7, 141.8, 136.8, 123.0, 120.8, 111.1, 111.1, 107.3, 55.1, 53.0, 42.8, 28.5, 27.5, 11.7; IR (neat) ν: 2956.1, 2930.9 (C-H), 1658.9 (C=O),

1256.2 (C-O) cm^{-1}; HRMS (ESI) *m/z* calcd. for $C_{32}H_{41}N_4O_4$ $[M+H]^+$ 545.31278, found 545.31720.

1,1'-[[(1,1,3,3-Tetramethyldisiloxane-1,3-diyl)bis(propane-3,1-diyl)]bis(2-methyl-5-phenyl-1*H*-pyrrole-1,3-diyl)]bis(ethan-1-one) (1k)

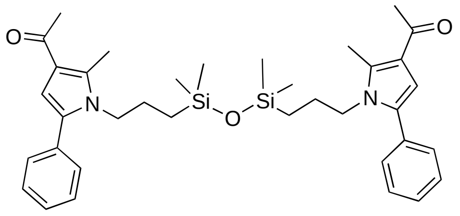

Prepared from acetophenone (1 mmol), 3,3'-(1,1,3,3-tetramethyldisiloxane-1,3-diyl)bis(propan-1-amine) (0.85 mmol) and acetylacetone (1.3 mmol); yield: 168 mg (55%); yellowish oil; ^1H NMR ($CDCl_3$, 250 MHz) δ 7.46-7.36 (m, 10H), 6.57 (s, 2H), 3.91 (m, 4H), 2.71 (s, 6H), 2.51 (s, 6H), 1.63-1.50 (m, 4H), 0.39-0.32 (m, 4H), 0.00 (s, 12H) ppm; ^{13}C NMR ($CDCl_3$, 63 MHz) δ 195.0, 135.6, 133.0, 132.9, 129.2, 128.4, 127.5, 120.7, 110.3, 46.6, 28.4, 24.5, 15.0, 12.0, 0.0 ppm; IR (neat) ν: 1650 (C=O), 1250 (C-O) cm^{-1}; Elemental analysis (%) calcd for $C_{36}H_{48}N_2O_3Si_2$: C 70.54, H 7.89, N, 4.57; found: C 70.52, H 7.82, N 4.51.

1,1'-[[(1,1,3,3-Tetramethyldisiloxane-1,3-diyl)bis(propane-3,1-diyl)]bis(2-methyl-5-(thiophen-2-yl)-1*H*-pyrrole-1,3-diyl)]bis(ethan-1-one) (1l)

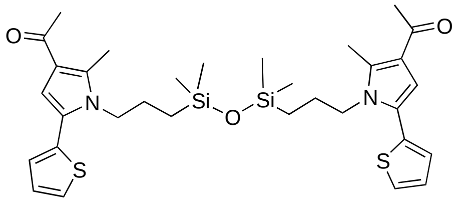

Prepared from 2-acetylthiophene (1 mmol), 1,3-bis(aminopropyl)tetramethyldisiloxane (0.85 mmol) and acetylacetone (1.3 mmol); yield: 187 mg (60%); dark yellow oil; ^1H NMR (250 MHz, $CDCl_3$) δ 7.35 – 7.32 (m, 2H), 7.09 – 7.06 (m, 2H), 7.03 – 7.01 (m, 2H), 6.63 (s, 2H), 3.92 – 3.86 (m, 4H), 2.62 (s, 6H), 2.43 (s, 6H), 1.67 – 1.54 (m, 4H), 0.45 – 0.38 (m, 4H), 0.00 (s, 12H); ^{13}C NMR (63 MHz, $CDCl_3$) δ 194.9, 136.3, 133.7, 127.3, 126.7, 125.8, 124.9, 120.8, 112.1, 46.8, 28.5, 24.8, 15.2, 12.0, 0.1; IR (neat) ν: 2952.1, 2926.9 (C-H), 1652.7 (C=O), 1252.5 (C-O) cm^{-1}; HRMS (ESI) *m/z* calcd. for $[M+H]^+$ 625.24101, found 625.24265; elemental analysis (%) calcd. for $C_{32}H_{44}N_2O_3S_2Si_2$: C, 61.50; H, 7.10; N, 4.48; found: C, 61.48; H, 7.06; N, 4.49.

Dimethyl 1,1'-[(1,1,3,3-tetramethyldisiloxane-1,3-diyl)bis(propane-3,1-diyl)]bis[5-(furan-2-yl)-2-methyl-1*H*-pyrrole-3-carboxylate] (1m)

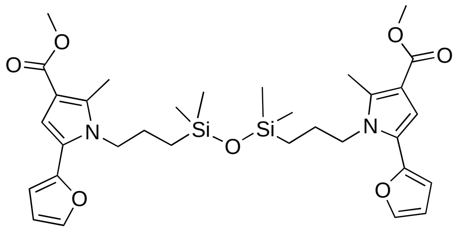

Prepared from 2-furyl methyl ketone (1 mmol), 1,3-bis(aminopropyl)tetramethyldisiloxane (0.85 mmol) and methyl acetoacetate (1.3 mmol); yield: 187 mg (65%); dark yellow oil; ^{1}H NMR (250 MHz, $CDCl_3$) δ 7.42 – 7.41 (m, 2H), 6.74 (s, 2H), 6.44 – 6.42 (m, 2H), 6.34 (dd, *J* = 3.3, 0.8 Hz, 2H), 3.97 – 3.90 (m, 4H), 3.81 (s, 6H), 2.57 (s, 6H), 1.69 – 1.56 (m, 6H), 0.47 – 0.40 (m, 4H), 0.00 (s, 12H); ^{13}C NMR (63 MHz, $CDCl_3$ δ 194.9, 136.3, 133.7, 127.3, 126.7, 125.8, 124.9, 120.8, 112.1, 46.8, 28.5, 24.8, 15.2, 12.0, 0.1; IR (neat) ν: 2950.9 (C-H), 1705.4 (C=O), 1252.1 (C-O) cm^{-1}; elemental analysis (%) calcd. for $C_{32}H_{44}N_2O_7Si_2$: C, 61.51; H, 7.10; N, 4.48; found: C, 61.48; H, 7.05; N, 4.48.

Diethyl 1,1'-[(1,1,3,3-tetramethyldisiloxane-1,3-diyl)bis(propane-3,1-diyl)]bis[2-(2-ethoxy-2-oxoethyl)-5-phenyl-1*H*-pyrrole-3-carboxylate] (1n)

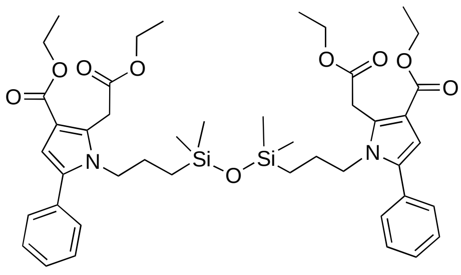

Prepared from acetophenone (0.5 mmol), 1,3-bis(aminopropyl)tetramethyldisiloxane (0.85 mmol) and diethyl 3-oxopentanedioate (1.3 mmol); yield: 237 mg (58%); yellowish oil; ^{1}H NMR (250 MHz, $CDCl_3$) δ 7.53 – 7.41 (m, 10H), 6.74 (s, 2H), 4.46 – 4.28 (m, 8H), 4.29 (s, 4H), 4.09 – 3.89 (m, 4H), 1.59 – 1.39 (m, 16H), 0.51 – 0.28 (m, 4H), 0.00 (s, 12H); ^{13}C NMR (63 MHz, $CDCl_3$) δ 170.0, 165.1, 134.1, 132.9, 131.5, 129.2, 128.4, 127.6, 113.4, 110.2, 61.0, 59.4, 47.2, 31.5, 24.8, 15.0, 14.4, 14.1, -0.1; IR (neat) ν: 2953.6, 2933.9, 2900.7 (C-H), 1735.5, 1695.2 (C=O), 1242.4 (C-O) cm^{-1}; elemental analysis (%) calcd. for $C_{44}H_{60}N_2O_9Si_2$: C, 64.67; H, 7.40; N, 3.43; found: C, 64.68; H, 7.37; N, 3.40.

Dimethyl 1,1'-[(1,1,3,3-tetramethyldisiloxane-1,3-diyl)bis(propane-3,1-diyl)]bis(2-methyl-1,4-dihydroindeno[1,2-*b*]pyrrole-3-carboxylate) (1o)

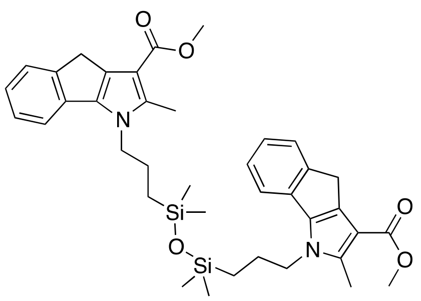

Prepared from 1-indanone (1 mmol), 1,3-bis(aminopropyl)tetramethyldisiloxane (0.85 mmol) and methyl acetoacetate (1.3 mmol); yield: 200 mg (66%); dark yellow oil; ^{1}H NMR (250 MHz, $CDCl_3$) δ 7.47 (d, *J* = 7.3 Hz, 2H), 7.29 – 7.20 (m, 4H), 7.13 – 7.06 (m, 2H), 4.04 (m, 4H), 3.86 (s, 6H), 3.63 (s, 4H), 2.62 (s, 6H), 1.84 – 1.69 (m, 4H), 0.61 – 0.48 (m, 4H),

0.00 (s, 12H); ^{13}C NMR (63 MHz, $CDCl_3$) δ 166.2, 146.7, 139.2, 136.4, 135.2, 129.6, 126.3, 125.3, 123.0, 115.7, 107.9, 50.7, 47.8, 32.0, 24.8, 15.2, 11.4, 0.1; IR (neat) ν: 2948.6, 2900.2 (C-H), 1702.0 (C=O), 1280.5 (C-O) cm^{-1}; elemental analysis (%) calcd. for $C_{38}H_{48}N_2O_5Si_2$: C, 68.23; H, 7.23; N, 4.19; found: C, 68.17; H, 7.22; N, 4.14.

***N*,*N*,*N*-tris[2-(3-acetyl-5-phenyl-2-methyl-1*H*-pyrrol-1-yl)ethyl]amine (8)**

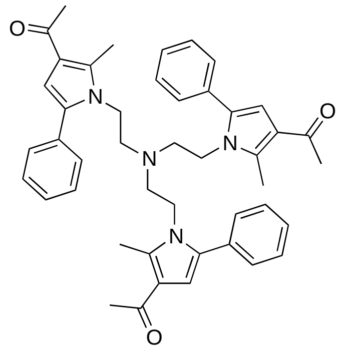

Following the general procedure, prepared from acetophenone (1.8 mmol), *N*,*N*-bis(2-aminoethyl)ethane-1,2-diamine (1.2 mmol) and acetylacetone (2.7 mmol). Purification by flash column chromatography on silica gel eluting with a gradient from dichloromethane to 98:02 dichloromethane: methanol afforded 145 mg (35%) of pyrrole **8** as a yellowish oil. ^{1}H NMR ($CDCl_3$, 250 MHz) χ 7.42-7.21 (m, 15H), 6.45 (s, 3H), 3.46 (t, 6H, *J* = 7.2 Hz), 2.42 (s, 9H), 2.39 (s, 9H), 2.07 (t, 6H, *J* = 7.2 Hz) ppm; ^{13}C NMR ($CDCl_3$, 63 MHz) δ 194.9, 135.4, 132.8, 132.5, 129.2, 128.6, 128.0, 120.9, 110.6, 54.2, 41.7, 28.5, 11.9 ppm; IR (neat) ν: 2973.6, 2927.4 (C-H), 1650.4 (C=O) cm^{-1}; Elemental analysis (%) calcd. for $C_{45}H_{48}N_4O_3$: C 78.00, H 6.98, N 8.09; found: C 77.91, H 6.93, N 8.05.

Representative intermediate bis-β-enaminone: Isolation of dimethyl 3,3'-(butane-1,4-diylbis(azanediyl))(2*Z*,2'*Z*)bis(but-2-enoate)

A mixture of 1,4-butanediamine (75 mg, 0.85 mmol), methyl acetoacetate (151 mg, 1.3 mmol) and cerium(IV) ammonium nitrate (71 mg, 0.13 mmol) in 0.5 mL of methanol was stirred at room temperature during 30 min, and the solvent was evaporated to give the crude enaminone as a white foamy solid. ^{1}H-NMR ($CDCl_3$, 300 MHz) δ 8.51 (s, 1H), 4.39 (s, 1H), 3.55 (s, 3H), 3.18 (d, *J* = 5.5 Hz, 2H), 1.85 (s, 3H), 1.60 – 1.58 (m, 2H) ppm. ^{13}C-NMR ($CDCl_3$, 75 MHz) δ 170.7, 161.6, 81.7, 49.7, 42.4, 27.5, 19.1 ppm.

3. General procedure for the synthesis of pyrrole derivatives 11 under solvent-free high-speed vibration milling (HSVM) conditions

The suitable α-iodoketone (0.5 mmol), prepared following procedure described in page S3, and a mixture of amine (1 mmol), the suitable β-dicarbonyl compound (0.75 mmol) and cerium(IV) ammonium nitrate (CAN, 5 mol %), previously stirred at room temperature during 30/60 min, and silver nitrate (0.5 mmol) were added to the vessel. The reaction was subjected to the vibratory movement at the same frequency for 60 min. Then, the reaction vessel was cleansed with ethyl acetate or dichloromethane and the suspension was filtered to remove the silver iodide precipitate. The organic

layer was washed with water (2 mL), dried over anhydrous sodium sulfate and the solvent was removed under reduced pressure. Purification by flash column chromatography on silica gel eluting with a gradient from petroleum ether to 8:2 petroleum ether–ethyl acetate afforded the desired pyrrole derivatives **11**.

Compound **11b** was prepared following the general procedure for compounds **1** (page S2).

Methyl 1-allyl-5-(furan-2-yl)-2-methyl-1*H*-pyrrole-3-carboxylate (11a)

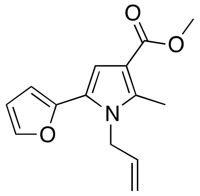

Prepared from 2-furyl methyl ketone (0.5 mmol), allylamine (1 mmol) and methyl acetoacetate (0.75 mmol); yield: 106 mg (87%); dark yellow oil; ^{1}H NMR (250 MHz, $CDCl_3$) δ 7.34 – 7.33 (m, 1H), 6.70 (s, 1H), 6.34 – 6.32 (m, 1H), 6.28 – 6.26 (m, 1H), 5.90 – 5.77 (m, 1H), 5.10 – 5.05 (m, 1H), 4.77 – 4.69 (m, 1H), 4.54 – 4.51 (m, 2H), 3.72 (m, 3H), 2.46 (s, 3H); ^{13}C NMR (63 MHz, $CDCl_3$) δ 165.7, 146.5, 141.7, 137.5, 132.9, 123.7, 116.3, 111.7, 111.0, 110.0, 107.0, 50.8, 46.9, 10.9; IR (neat) ν: 3124.9, 2947.4 (C-H), 1700.8 (C=O), 1244.1, 1200.2 (C-O) cm^{-1}; elemental analysis (%) calcd. for $C_{14}H_{15}NO_3$: C, 68.56; H, 6.16; N, 5.71; found: C, 68.59; H, 6.13; N, 5.76.

1-(1-Allyl-5-(*tert*-butyl)-2-methyl-1*H*-pyrrol-3-yl)ethan-1-one (11b)

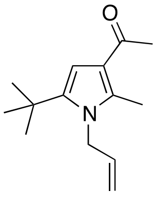

Prepared from 3,3-dimethylbutan-2-one (0.5 mmol), allylamine (1 mmol) and acetylacetone (0.75) ; yield: 65 mg (60%); green oil; ^{1}H NMR (250 MHz, $CDCl_3$) δ 6.28 (s, 1H), 6.02 – 5.77 (m, 1H), 5.24 – 5.16 (m, 1H), 4.76 – 4.62 (m, 3H), 2.50 (s, 3H), 2.41 (s, 3H), 1.36 (s, 9H); ^{13}C NMR (63 MHz, $CDCl_3$) δ ^{13}C NMR (63 MHz, $CDCl_3$) δ 195.1, 140.3, 137.0, 133.3, 119.5, 116.4, 106.4, 47.1, 31.8, 30.7, 28.6, 11.6; IR (neat) ν: 2964.7, 2869.9 (C-H), 1649.3 (C=O) cm^{-1}; elemental analysis (%) calcd. for $C_{14}H_{21}NO$: C, 76.67; H, 9.65; N, 6.39; found: C, 76.76; H, 9.64; N, 6.37.

Methyl 1-(but-3-en-1-yl)-2-methyl-5-(thiophen-2-yl)-1*H*-pyrrole-3-carboxylate (11c)

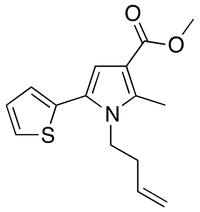

Prepared from 2-acetylthiophene (0.5 mmol), 3-buten-1-amine (1 mmol) and methyl acetoacetate (0.75 mmol); yield: 98 mg (71%); green oil; ^{1}H NMR (250 MHz, $CDCl_3$) δ 7.10 – 7.07 (m, 1H), 6.86 – 6.80 (m, 2H), 6.45 (s, 1H), 5.53 – 5.37 (m, 1H), 4.85 – 4.78 (m, 2H), 3.81 – 3.75 (m, 2H), 3.58 (s, 3H), 2.39 (s, 3H), 2.17 – 2.09 (m, 2H); ^{13}C NMR (63 MHz, $CDCl_3$) δ 165.6, 137.0, 133.7, 133.4, 127.2, 126.4, 125.6, 126.0, 117.5, 111.6, 111.4, 50.7, 43.5, 34.7, 11.4; IR (neat) ν: 2977.8, 2945.2, 2849.5 (C-H), 1698.0 (C=O), 1240.1, 1188.3 (C-O) cm^{-1}; elemental analysis

(%) calcd. for $C_{15}H_{17}NO_2S$: C, 65.43; H, 6.22; N, 5.09; found: C, 65.45; H, 6.29; N, 5.06.

Ethyl 1-allyl-2-(2-ethoxy-2-oxoethyl)-5-(furan-2-yl)-1*H*-pyrrole-3-carboxylate (11d)

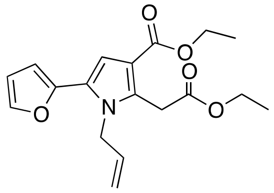

Prepared from 2-furyl methyl ketone (0.5 mmol), allylamine (1 mmol) and diethyl 1,3-acetonedicarboxylate (0.75 mmol); yield: 136 mg (80%); dark orange oil; ^{1}H NMR (250 MHz, $CDCl_3$) δ 7.46 (d, *J* = 1.8 Hz, 1H), 6.87 (s, 1H), 6.46 (dd, *J* = 3.3, 1.8 Hz, 1H), 6.41 (d, *J* = 3.3 Hz, 1H), 6.07 – 5.86 (m, 1H), 5.24 – 5.14 (m, 1H), 4.90 – 4.82 (m, 1H), 4.71 – 4.69 (m, 2H), 4.30 (q, *J* = 7.1 Hz, 2H), 4.18 (q, *J* = 7.1 Hz, 2H), 4.12 (s, 2H), 1.36 (t, *J* = 7.1 Hz, 3H), 1.28 (t, *J* = 7.1 Hz, 3H); ^{13}C NMR (63 MHz, $CDCl_3$) δ 169.9, 164.8, 146.3, 141.9, 133.1, 132.7, 124.7, 116.5, 114.0, 111.1, 110.4, 107.5, 61.2, 59.7, 47.2, 31.2, 14.4, 14.1; IR (neat) ν: 2980.5, 2924.4, 2852.3 (C-H), 1734.7, 1704.3 (C=O), 1245.7, 1181.3 (C-O) cm^{-1}; elemental analysis (%) calcd. for $C_{18}H_{21}NO_5$: C, 65.24; H, 6.39; N, 4.23; found: C, 65.27; H, 6.34; N, 4.19.

4. Preparation of compounds 12 by cross-metathesis reactions

A round-bottomed flask was charged with second generation Hoveyda–Grubbs catalyst (2 mmol %), CuI (2 mmol %) and the suitable pyrrole **11** (0.15 mmol) in dry diethyl ether (2 mL), stirred and heated at 40 °C during 16 (for compounds **12a**, **12c** and **12d**) or 48 hours (for compound **12b**). After completion of the reaction (controlled by TLC) the solvent was removed under reduced pressure and the residue was purified by flash column chromatography on silica gel eluting with a gradient from petroleum ether to 8:2 petroleum ether–ethyl acetate, affording the desired pyrroles **12**.

Dimethyl 1,1'-[(*E*) but-2-ene-1,4-diyl]bis[5-(furan-2-yl)-2-methyl-1*H*-pyrrole-3-carboxylate] (12a)

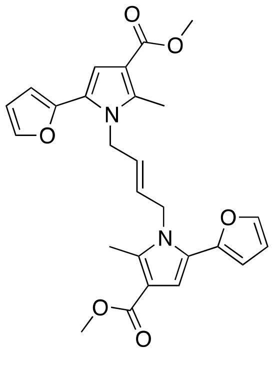

Prepared from compound **11a** (0.25 mmol); yield: 52 mg (88%); dark yellow solid; mp: 170-172 °C; ; ^{1}H NMR (250 MHz, $CDCl_3$) δ 7.40 – 7.39 (m, 2H), 6.80 (s, 2H), 6.42 – 6.41 (m, 2H), 6.30 – 6.29 (m, 2H), 5.22 (t, *J* = 1.6 Hz, 2H), 4.55 (d, *J* = 1.6 Hz, 4H), 3.84 (s, 6H), 2.51 (s, 6H); ^{13}C NMR (63 MHz, $CDCl_3$) δ 165.6, 146.3, 141.7, 137.2, 126.4, 123.6, 112.1, 111.0, 110.1, 107.0, 50.87, 45.6, 10.9; IR (neat) ν: 3011.6, 2944.7, 2849.5 (C-H), 1694.7 (C=O), 1248.4 (C-O), cm^{-1}; HRMS (ESI) *m/z* calcd. for $[M+Na]^+$ 485.16886, found 485.17047; elemental analysis (%) calcd. for $C_{26}H_{26}N_2O_6$: C, 67.52; H, 5.67; N, 6.06; found: C, 67.53; H, 5.61; N, 6.03.

(*E*)-1,1'-(But-2-ene-1,4-diyl)bis[5-(*tert*-butyl)-2-methyl-1*H*-pyrrole-1,3-diyl)]bis(ethan-1-one) (12b)

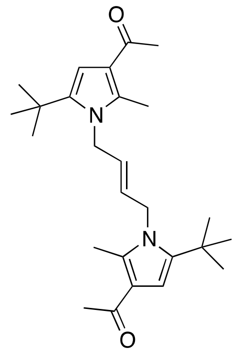

Prepared from compound **11b** (0.25 mmol); yield: 31 mg (70%); green oil; ^{1}H NMR (250 MHz, CDCl$_3$) δ 6.27 (s, 2H), 4.98 (br s, 2H), 4.59 (br s, 4H), 2.44 (s, 6H), 2.41 (s, 6H), 1.32 (s, 18H); ^{13}C NMR (63 MHz, CDCl$_3$) δ 195.2, 140.3, 136.5, 126.8, 119.7, 106.5, 45.9, 31.8, 30.6, 28.6, 11.4; IR (neat) ν: 2964.4, 2926.1, 2869.9 (C-H), 1650.4 (C=O), 1245.4 (C-O) cm^{-1}; elemental analysis (%) calcd. for $C_{26}H_{38}N_2O_2$: 76.06; H, 9.33; N, 6.82; found: C, 76.02; H, 9.26; N, 6.79.

(*E*,*Z*) Dimethyl 1,1'-(hex-3-ene-1,6-diyl)bis[2-methyl-5-(thiophen-2-yl)-1*H*-pyrrole-3-carboxylate] (12c)

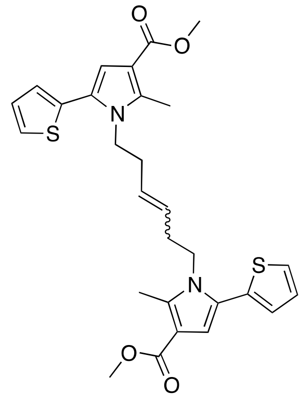

The reaction from compound **11c** (0.25 mmol) afforded a 1:1 mixture of the *E* and *Z* isomers; yield: 53 mg (80%); green oil. Only one of the isomers could be isolated in pure state, by recrystallization from methanol, and its data are the following: ^{1}H NMR (250 MHz, CDCl$_3$) δ 7.36 – 7.33 (m, 2H), 7.11 – 7.07 (m, 2H), 7.02 – 7.01 (m, 2H), 6.67 (s, 2H), 5.28 – 5.25 (m, 2H), 3.98 – 3.92 (m, 4H), 3.83 (s, 6H), 2.59 (s, 6H), 2.28 – 2.25 (m, 4H); ^{13}C NMR (63 MHz, CDCl$_3$) δ 165.7, 137.0, 133.8, 128.4, 127.3, 126.6, 125.7, 125.3, 111.8, 111.6, 50.8, 43.7, 33.7, 11.6; IR (neat) ν: 3011.6, 29944.7, 2849.5 (C-H), 1694.7 (C=O), 1241.8 (C-O) cm^{-1}; elemental analysis (%) calcd. for $C_{28}H_{30}N_2O_4S_2$: C, 64.34; H, 5.79; N, 5.36; found: C, 64.29: H, 5.80: N, 5.39.

Diethyl 1,1'-[(*E*) (but-2-ene-1,4-diyl)]bis[2-(2-ethoxy-2-oxoethyl)-5-(furan-2-yl)-1*H*-pyrrole-3-carboxylate] (12d)

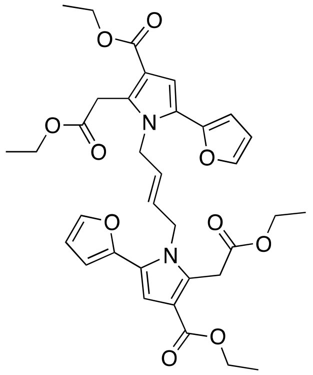

Prepared from compound **11d** (0.25 mmol); yield: 70 mg (88%); dark orange oil; ^{1}H NMR (250 MHz, CDCl$_3$) δ 7.43 – 7.42 (m, 2H), 6.85 (s, 2H), 6.44 – 6.42 (m, 2H), 6.34 – 6.33 (m, 2H), 5.26 (t, *J* = 1.6 Hz, 2H), 4.60 (br s, 4H), 4.30 (q, *J* = 7.1 Hz, 4H), 4.16 (q, *J* = 7.1 Hz, 4H), 4.02 (s, 4H), 1.37 (t, *J* = 7.1 Hz, 6H), 1.28 (d, *J* = 7.1 Hz, 6H); ^{13}C NMR (63 MHz, CDCl$_3$) δ 169.7, 164.7, 146.1, 141.9, 132.5, 126.8, 124.6, 114.1, 111.1, 110.4, 107.5, 61.2, 59.7, 45.9, 31.0, 14.4, 14.2; IR (neat) ν: 2973.6, 2927.4 (C-H), 1727.8, 1694.1 (C=O), 1249.8, 1225.6 (C-O) cm^{-1}; elemental analysis (%) calcd. for $C_{34}H_{38}N_2O_{10}$: C, 64.34; H, 6.04; N, 4.41; found: C, 64.31; H, 6.00; N, 4.38.

5. Copies of ^{1}H, ^{13}C and ^{19}F NMR spectra

Diethyl 1,1'-(ethane-1,2-diyl)bis(2-methyl-5-phenyl-1*H*-pyrrole-3-carboxylate) (1a)

^{1}H NMR (250 MHz, $CDCl_3$)

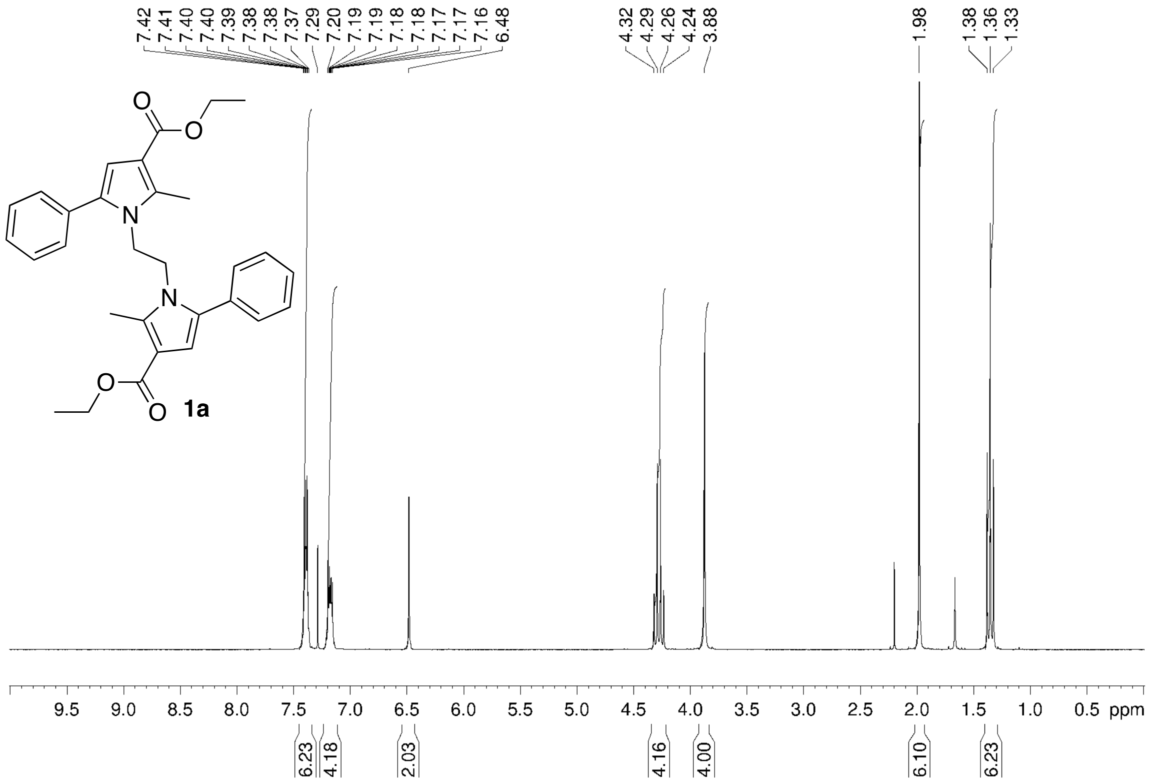

^{13}C NMR (63 MHz, $CDCl_3$)

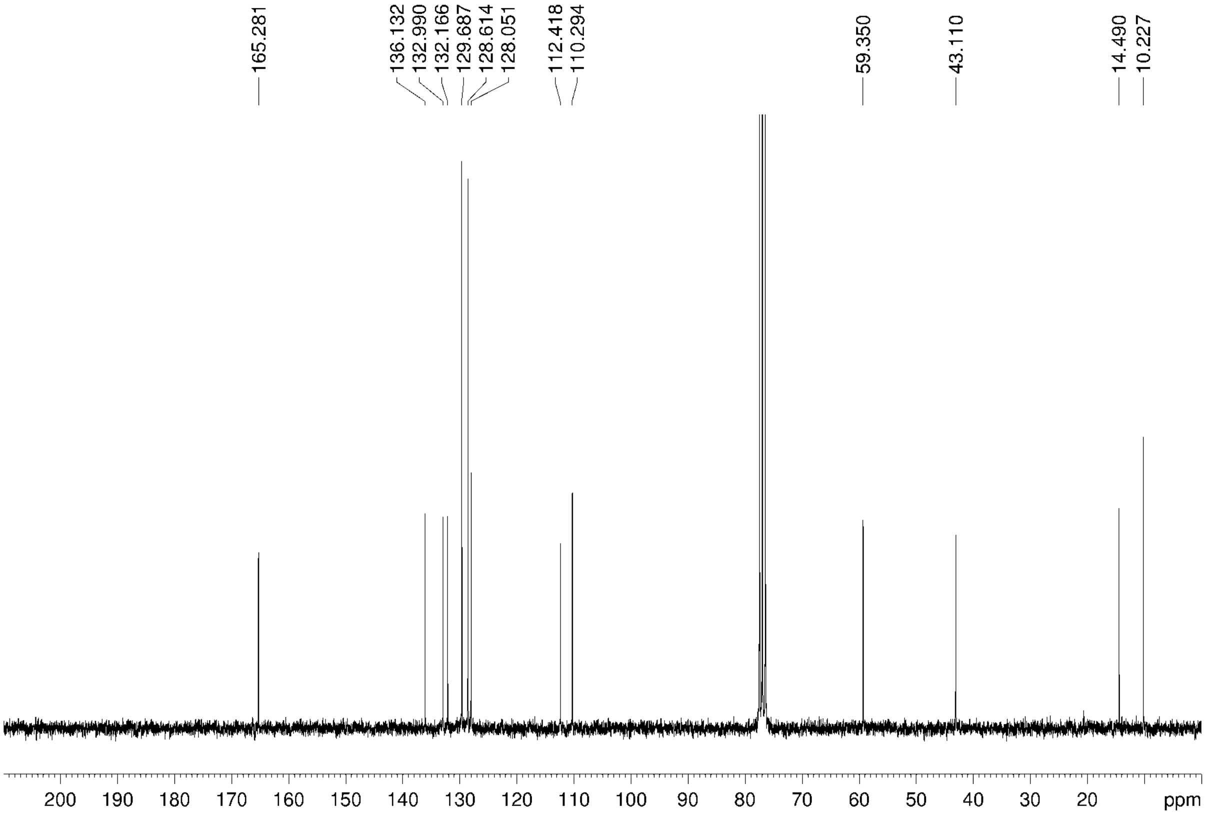

Dimethyl 1,1'-(propane-1,3-diyl)bis[5-(4-chlorophenyl)-2-methyl-1*H*-pyrrole-3-carboxylate] (1b)

^{1}H NMR (250 MHz, $CDCl_3$)

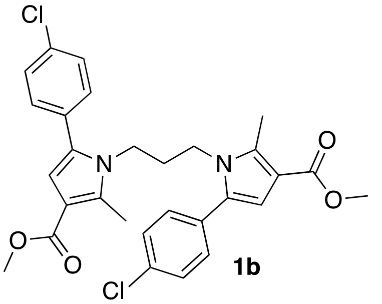

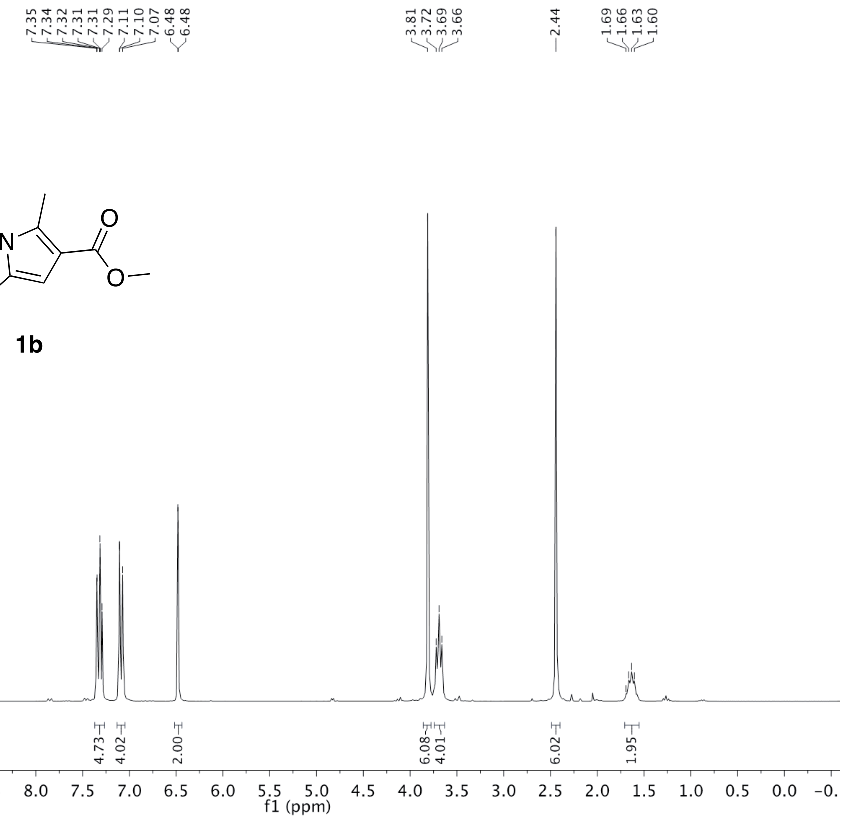

^{13}C NMR (63 MHz, $CDCl_3$)

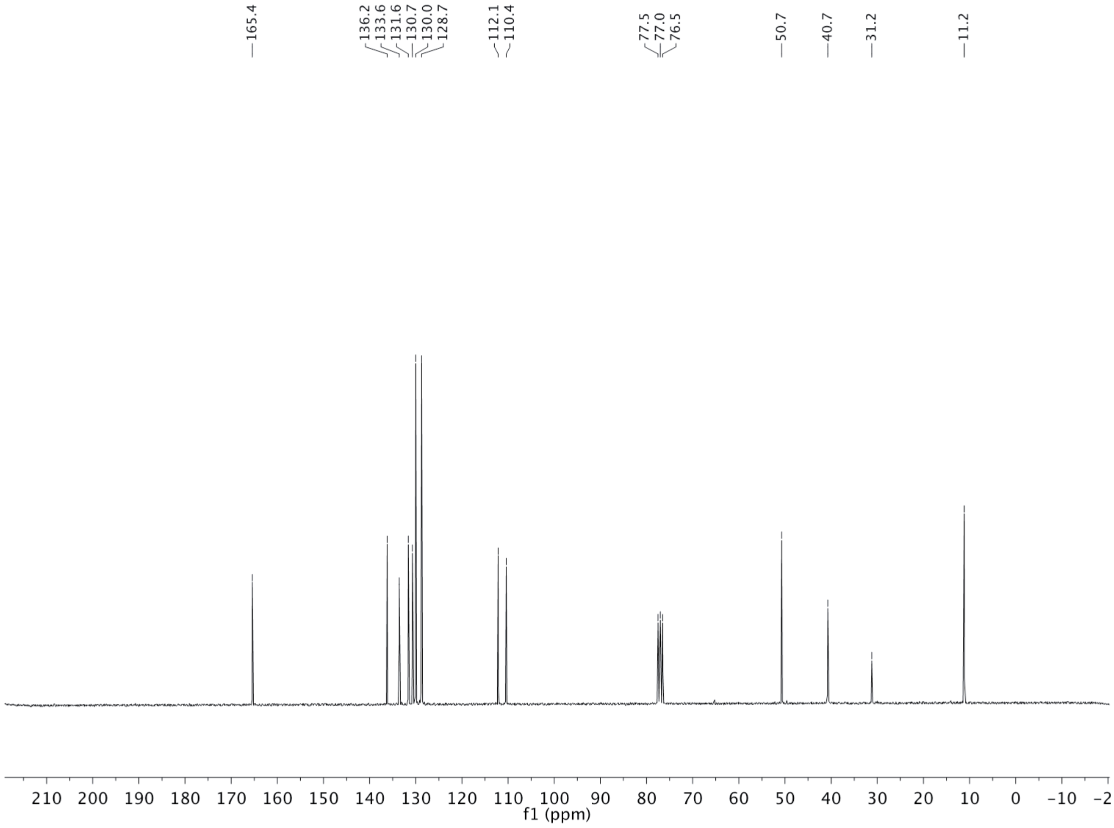

Dimethyl 1,1'-(butane-1,4-diyl)bis(2-methyl-5-phenyl-1*H*-pyrrole-3-carboxylate) (1c)

^{1}H NMR (250 MHz, $CDCl_3$)

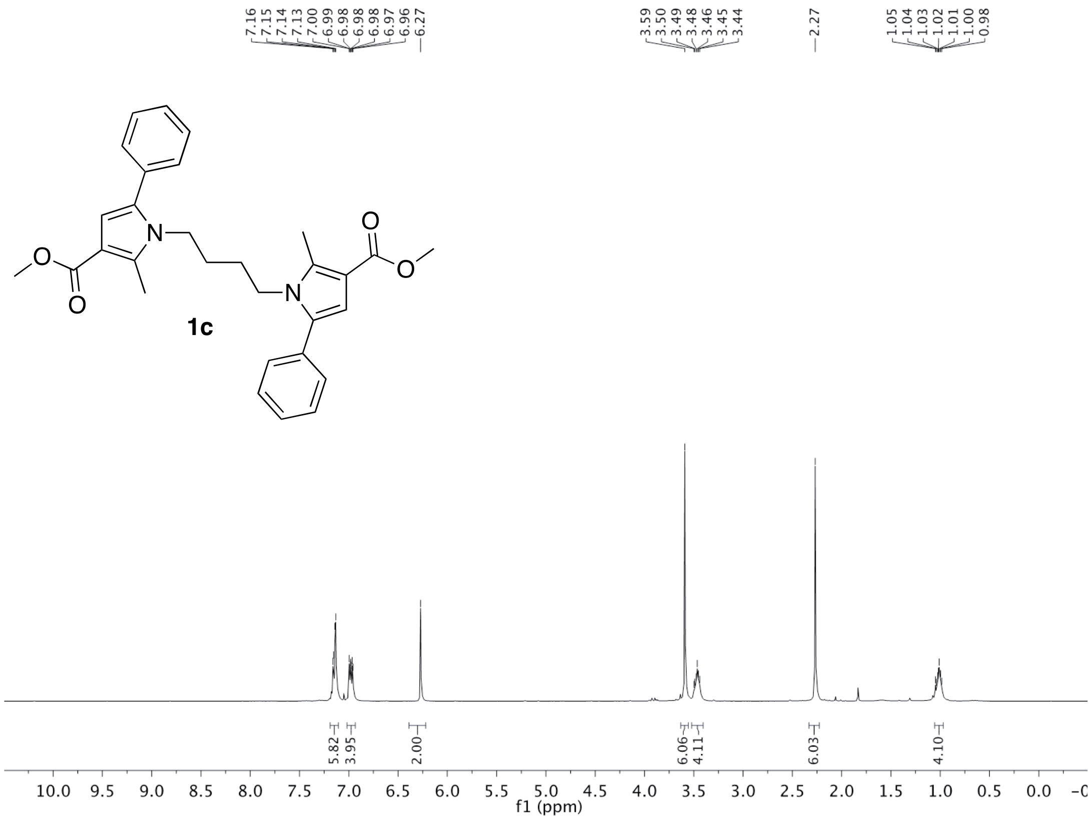

^{13}C NMR (63 MHz, $CDCl_3$)

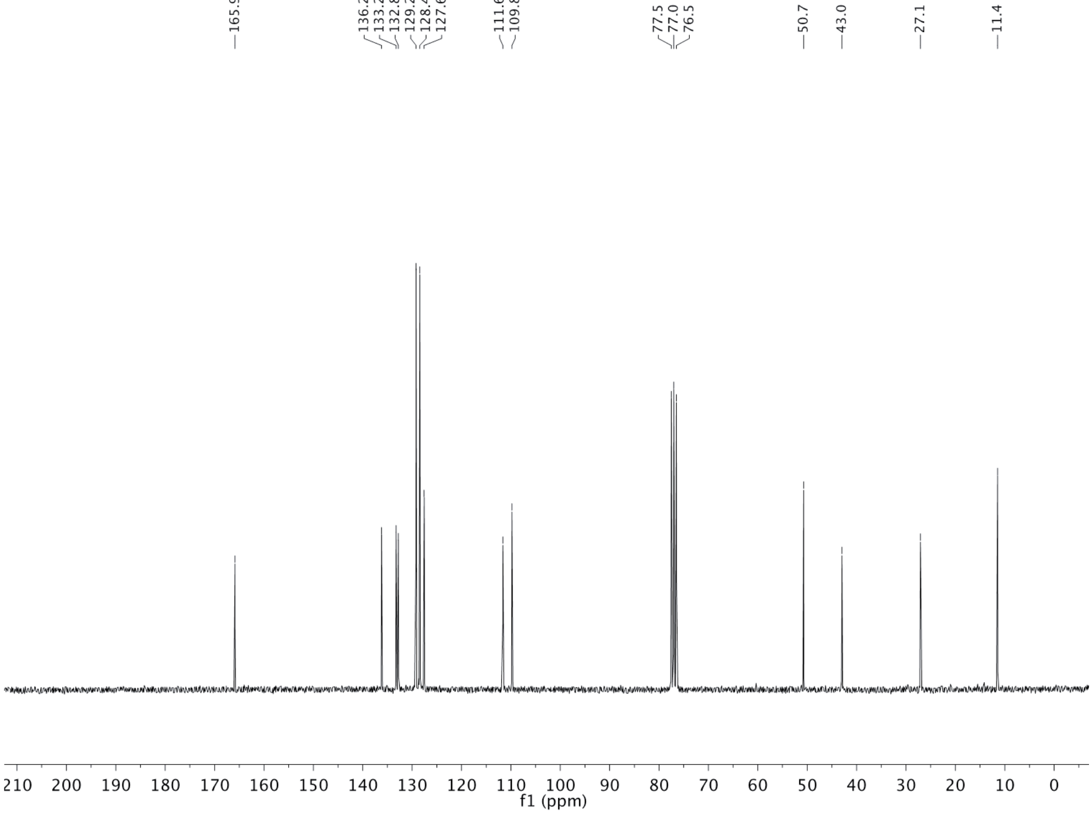

Dimethyl 1,1'-(butane-1,4-diyl)bis[5-(furan-2-yl)-2-methyl-1*H*-pyrrole-3-carboxylate] (1d)

^{1}H NMR (250 MHz, $CDCl_3$)

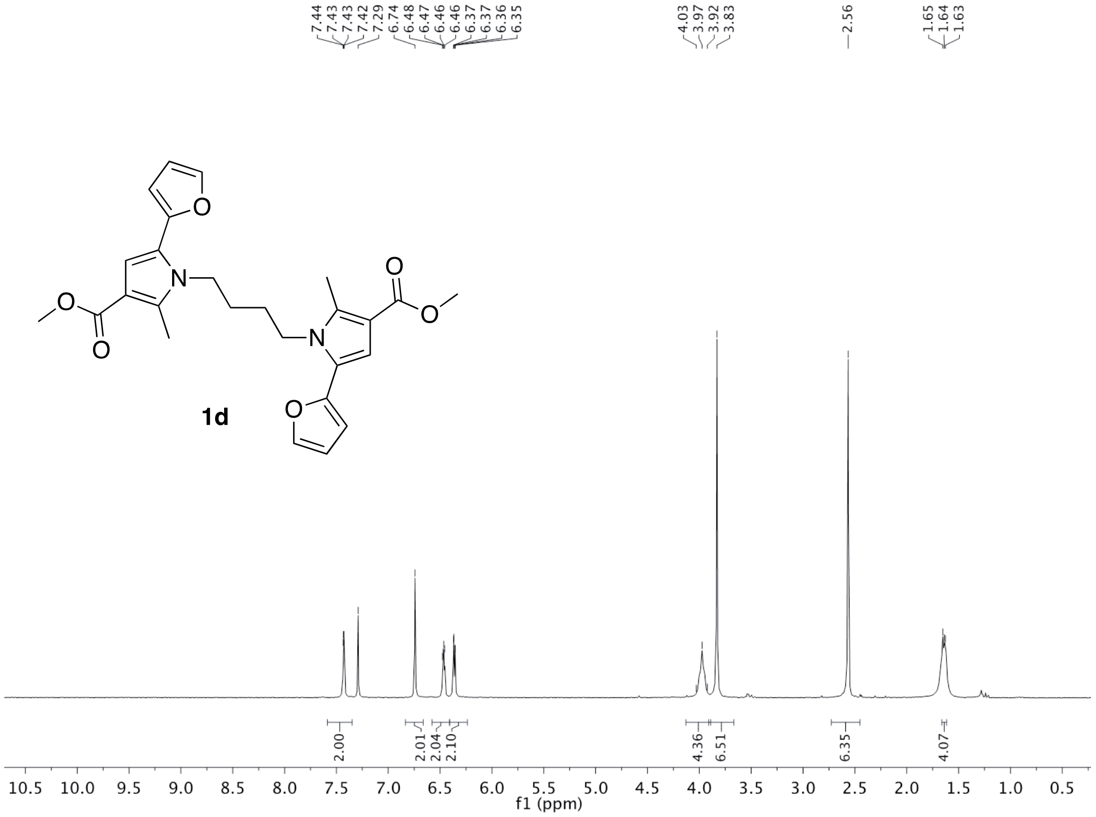

1d

^{13}C NMR (63 MHz, $CDCl_3$)

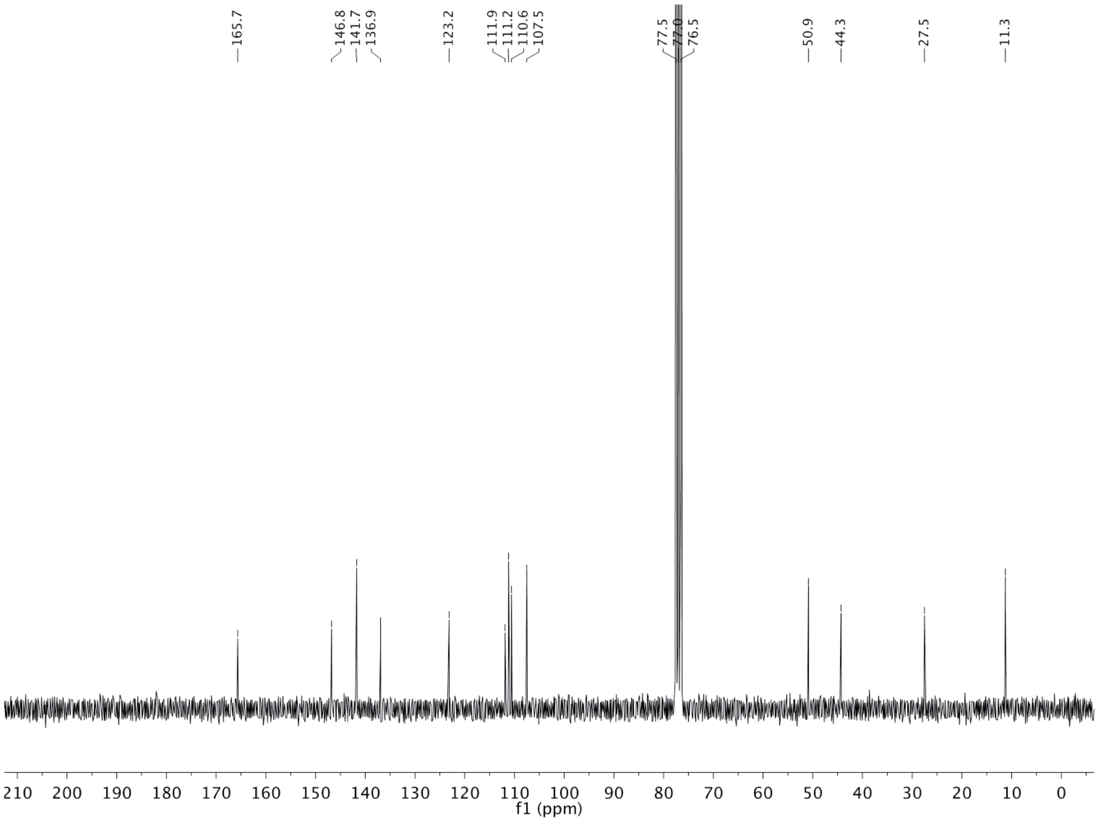

Dimethyl 1,1'-(heptane-1,7-diyl)bis(2-methyl-5-phenyl-1*H*-pyrrole-3-carboxylate) (1e)

^{1}H NMR (250 MHz, $CDCl_3$)

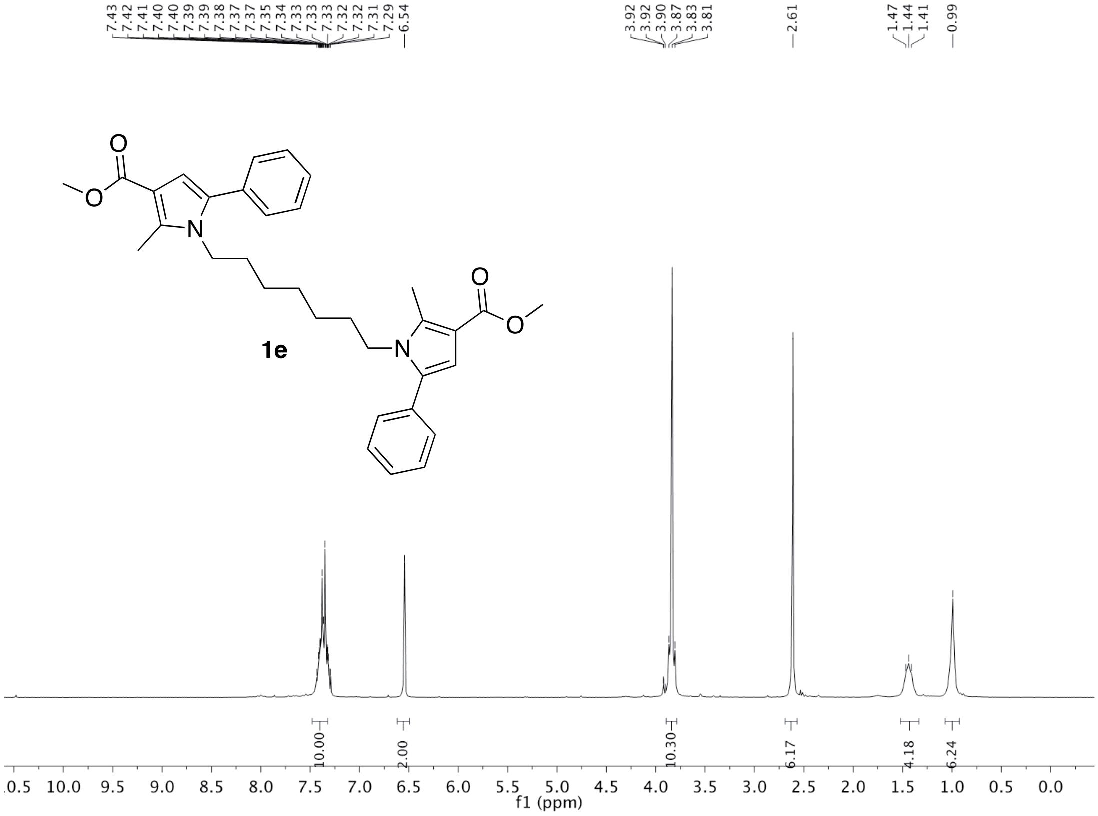

^{13}C NMR (63 MHz, $CDCl_3$)

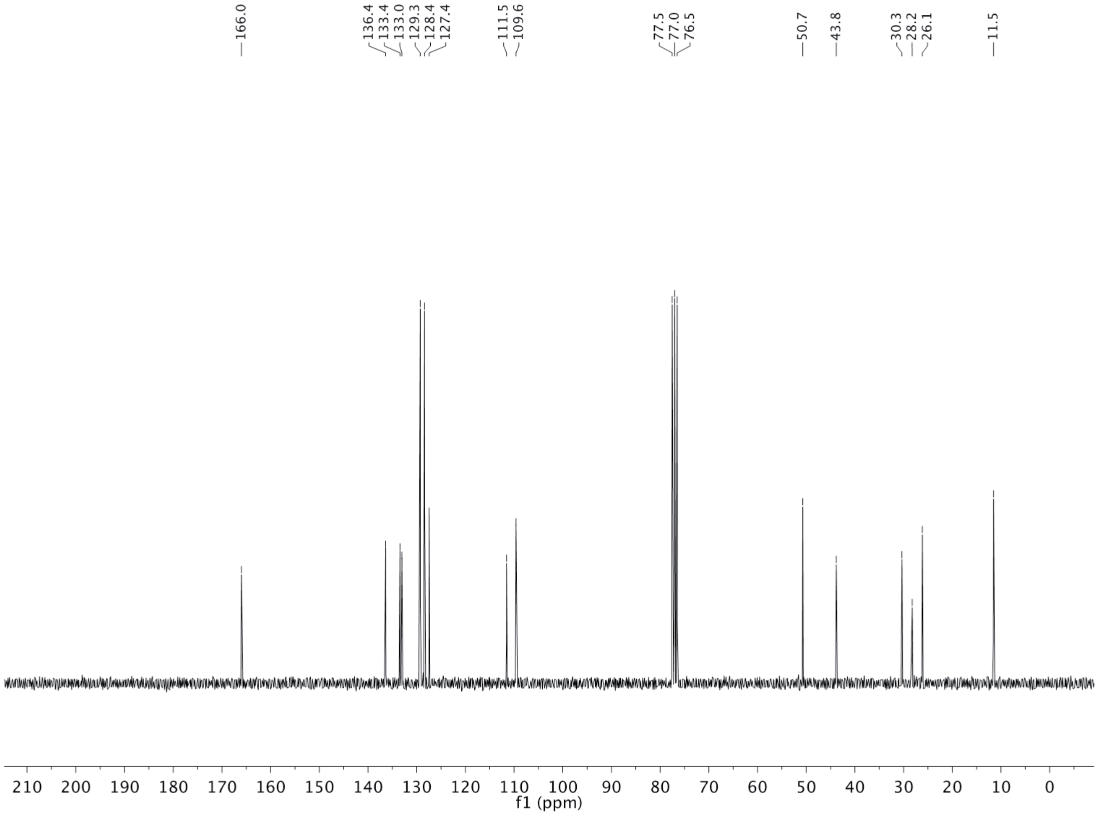

1,1'-(Octane-1,8-diyl)bis[5-(4-fluorophenyl)-2-methyl-1*H*-pyrrole-1,3-diyl]bis(ethan-1-one) (1f)

^{1}H NMR (250 MHz, $CDCl_3$)

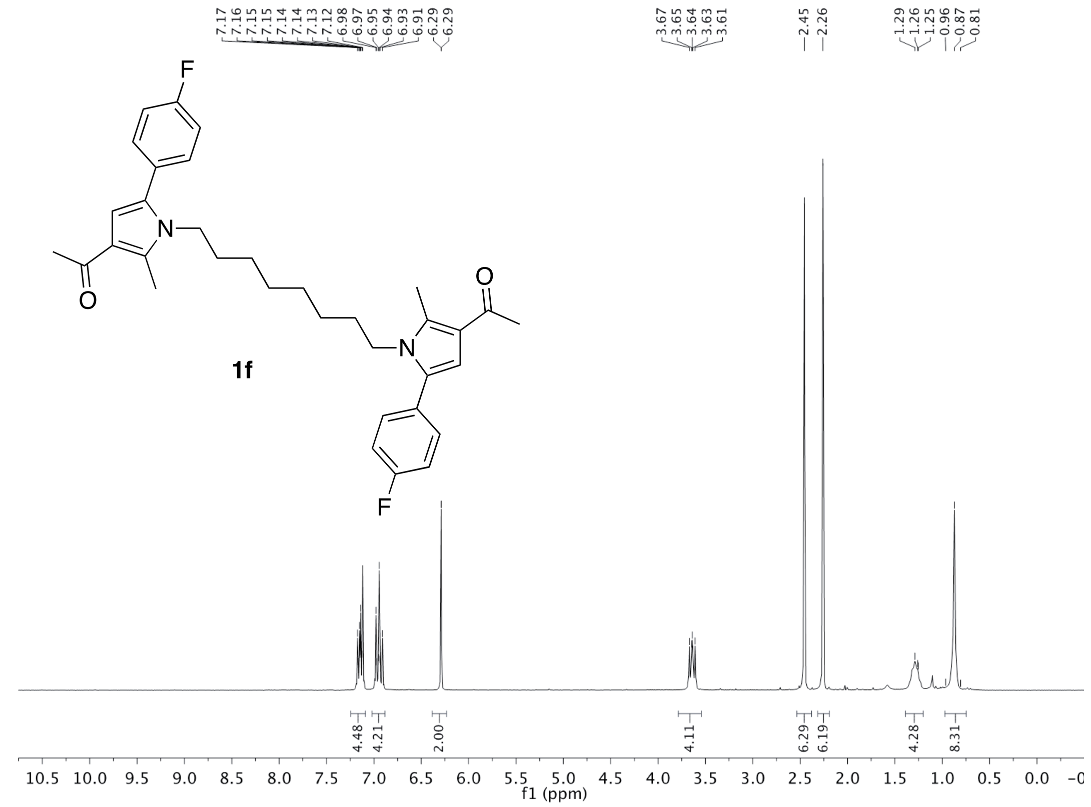

^{13}C NMR (63 MHz, $CDCl_3$)

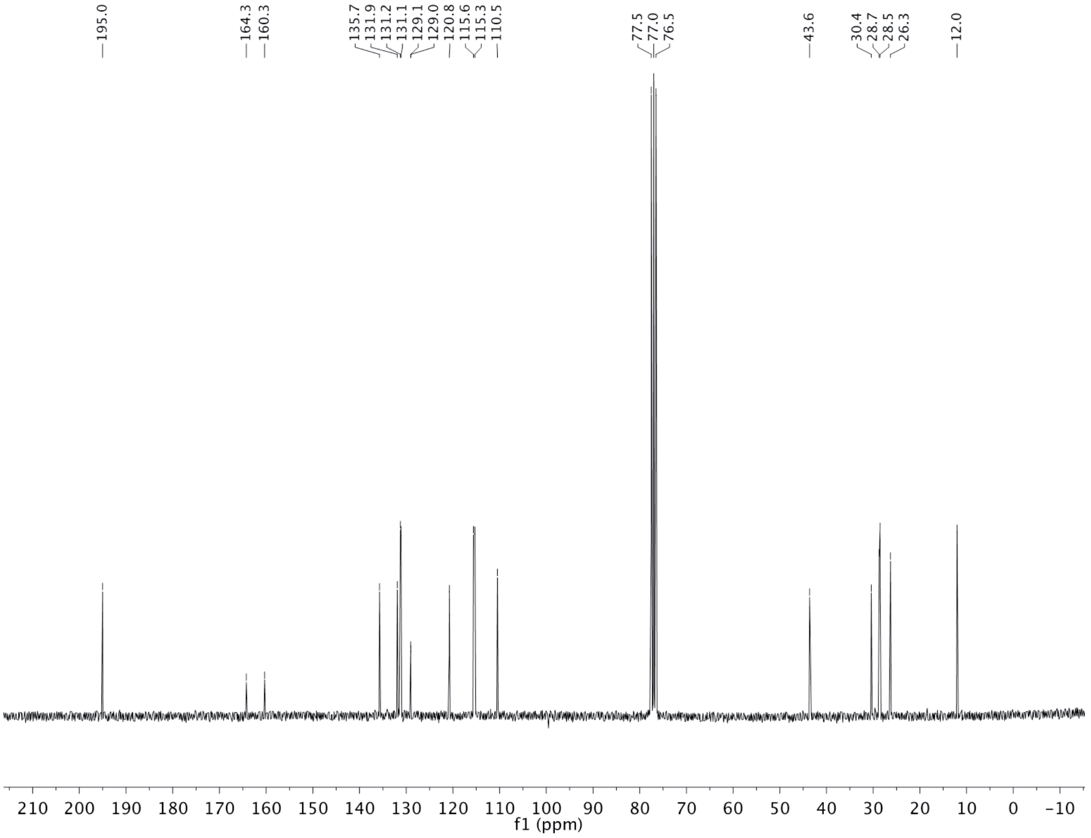

^{19}F NMR (235 MHz, $CDCl_3$)

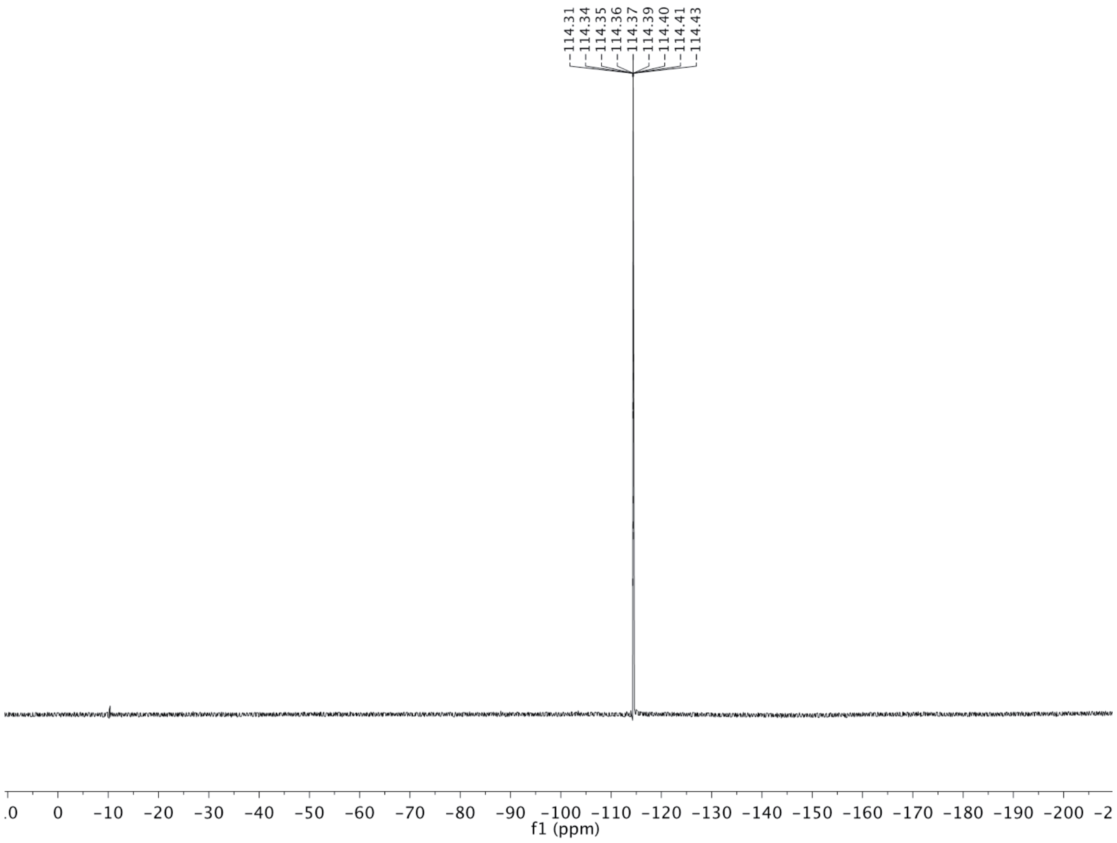

Dimethyl 1,1'-(dodecane-1,12-diyl)bis[5-(furan-2-yl)-2-(2-methoxy-2-oxoethyl)-1*H*-pyrrole-3-carboxylate] (1g)

^{1}H NMR (250 MHz, $CDCl_3$)

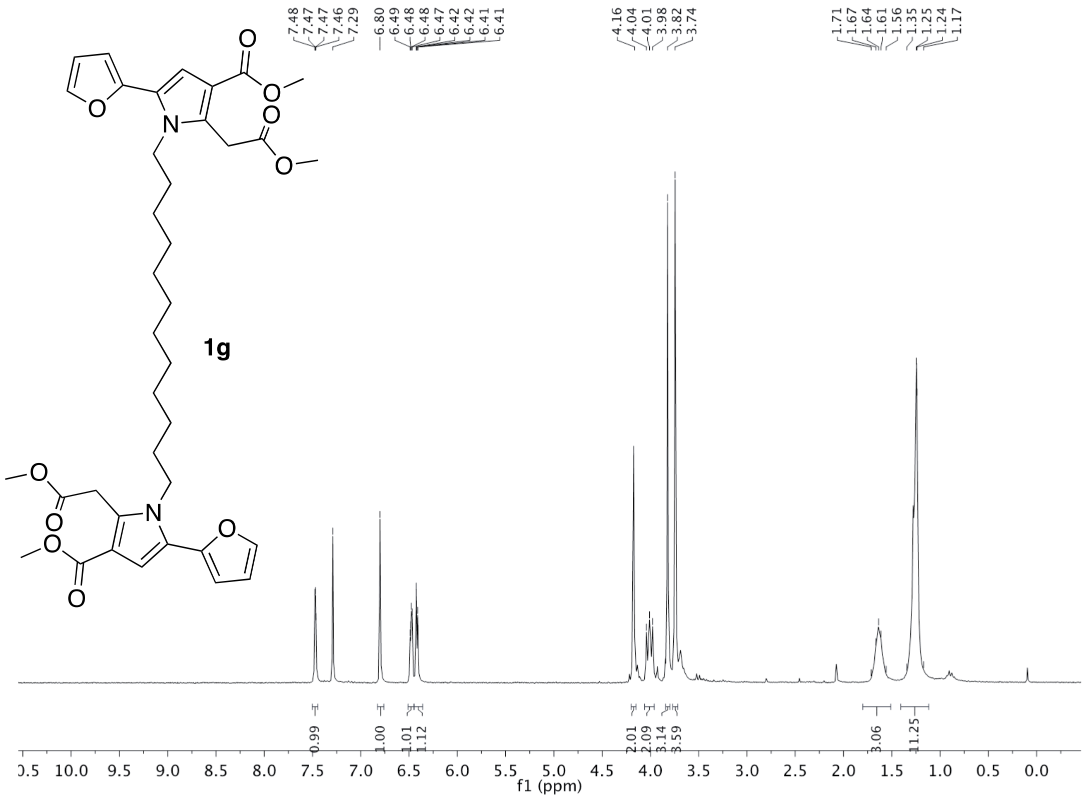

^{13}C NMR (63 MHz, $CDCl_3$)

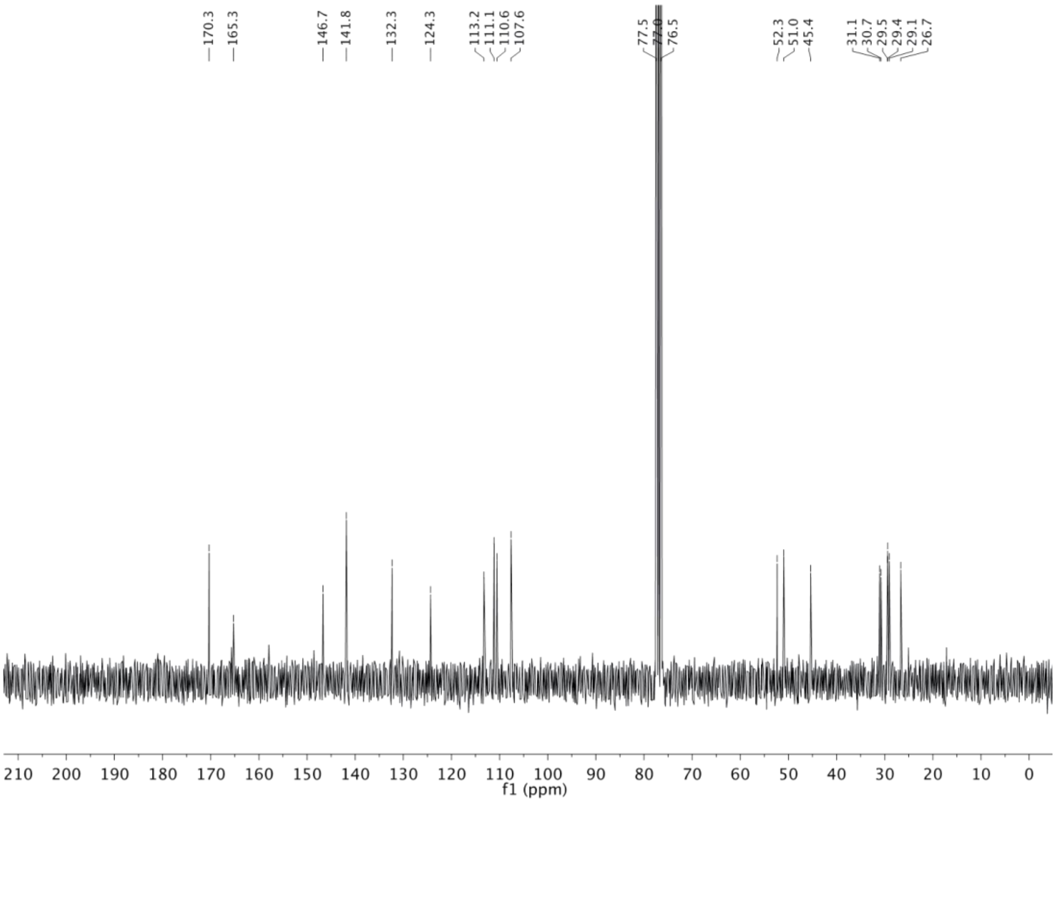

***N*,*N*-Bis[2-(3-ethoxycarbonyl-5-phenyl-2-methylpyrrol-1-yl)ethyl]amine (1h)**

^{1}H NMR (250 MHz, $CDCl_3$)

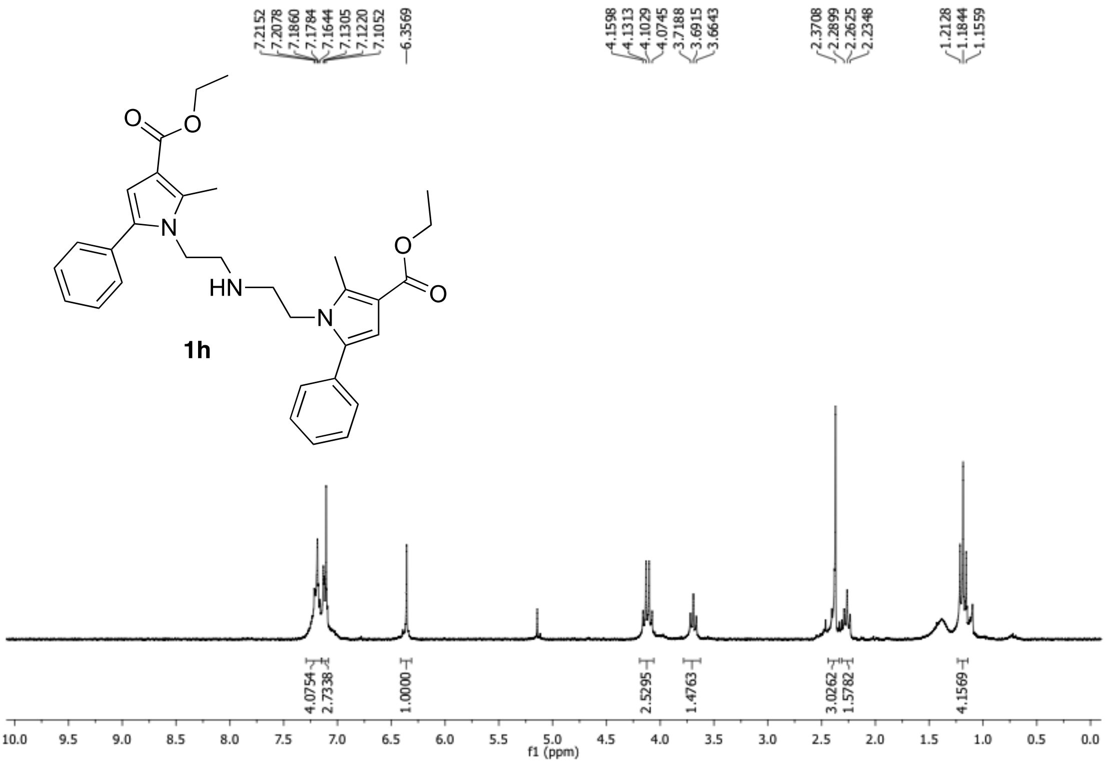

^{13}C NMR (63 MHz, $CDCl_3$) ^{1}H NMR (250 MHz, $CDCl_3$)

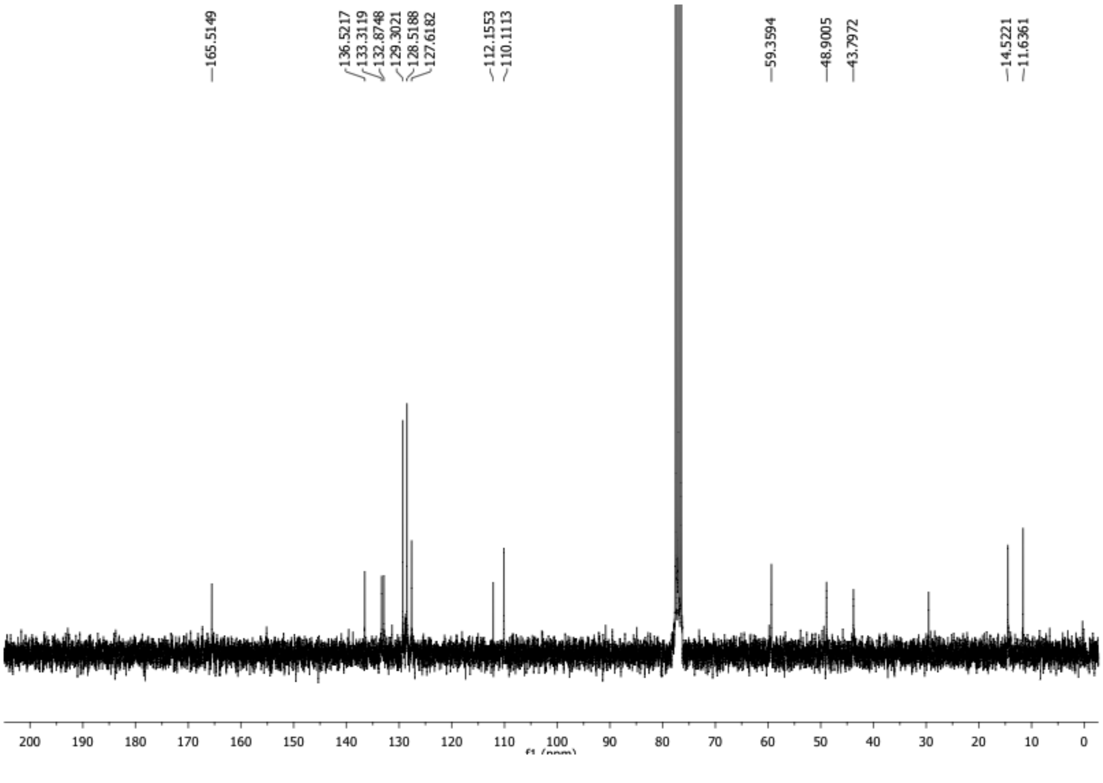

***N*1,*N*4-Bis[3-(3-ethoxycarbonyl-5-phenyl-2-methylpyrrol-1-yl)propyl]piperazine (1i)**

^{1}H NMR (250 MHz, $CDCl_3$)

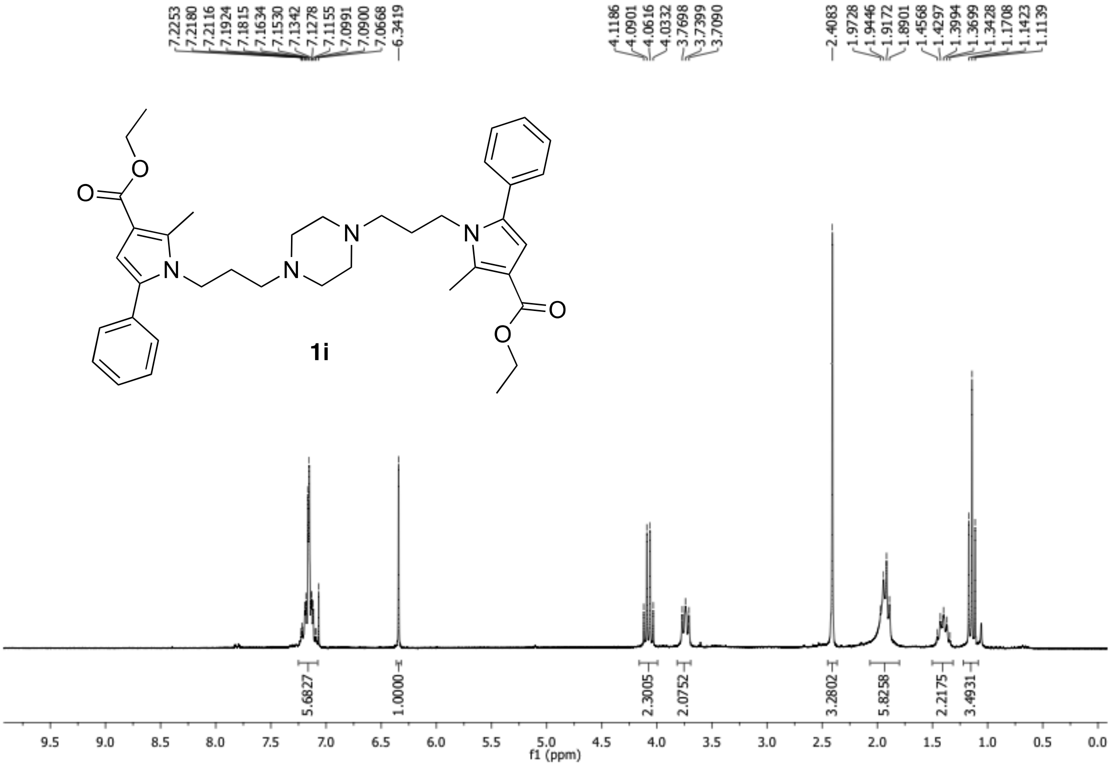

^{13}C NMR (63 MHz, $CDCl_3$)

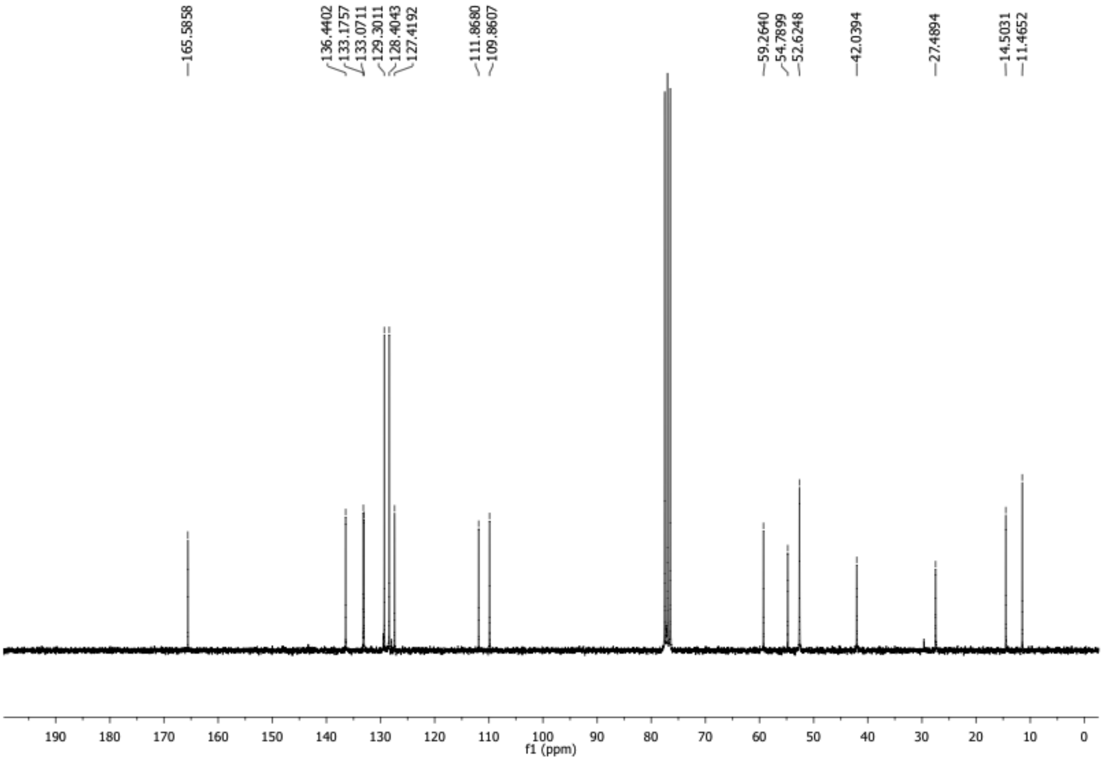

***N*1,*N*4-Bis[3-[3-(methylcarbonyl)-5-(furan-2-yl)-2-methylpyrrol-1-yl]propyl]piperazine (1j)**

^{1}H NMR (250 MHz, $CDCl_3$)

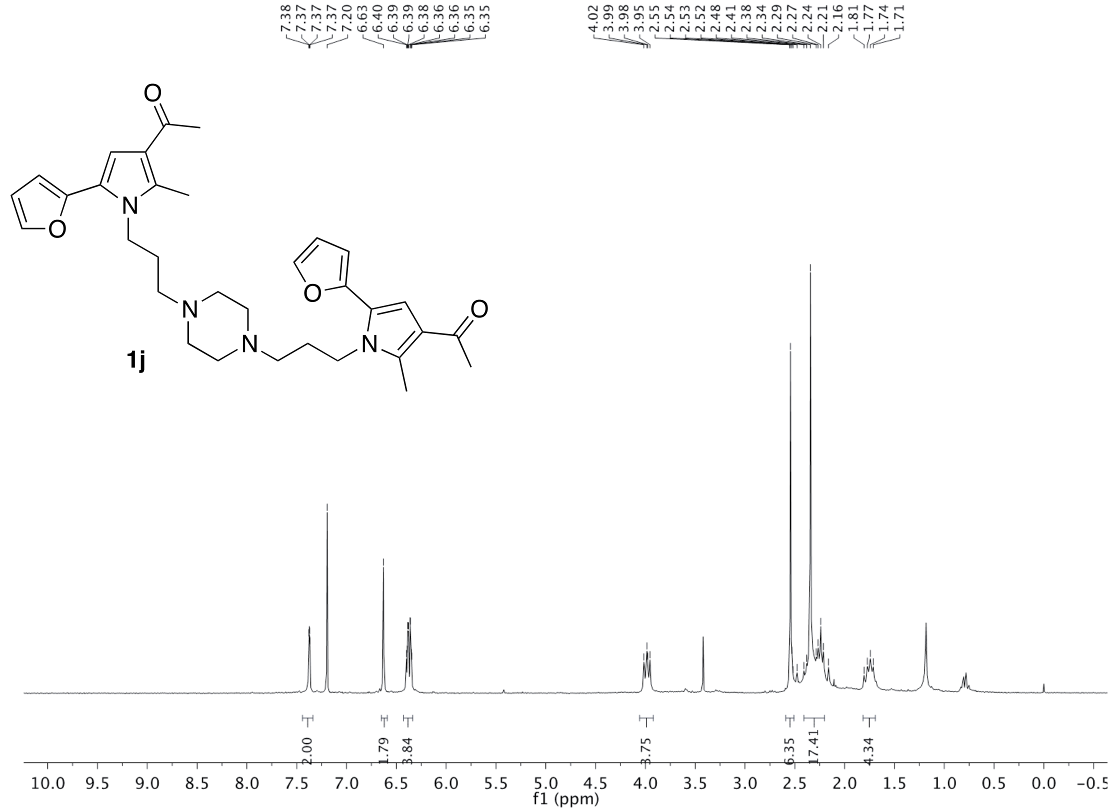

^{13}C NMR (63 MHz, $CDCl_3$)

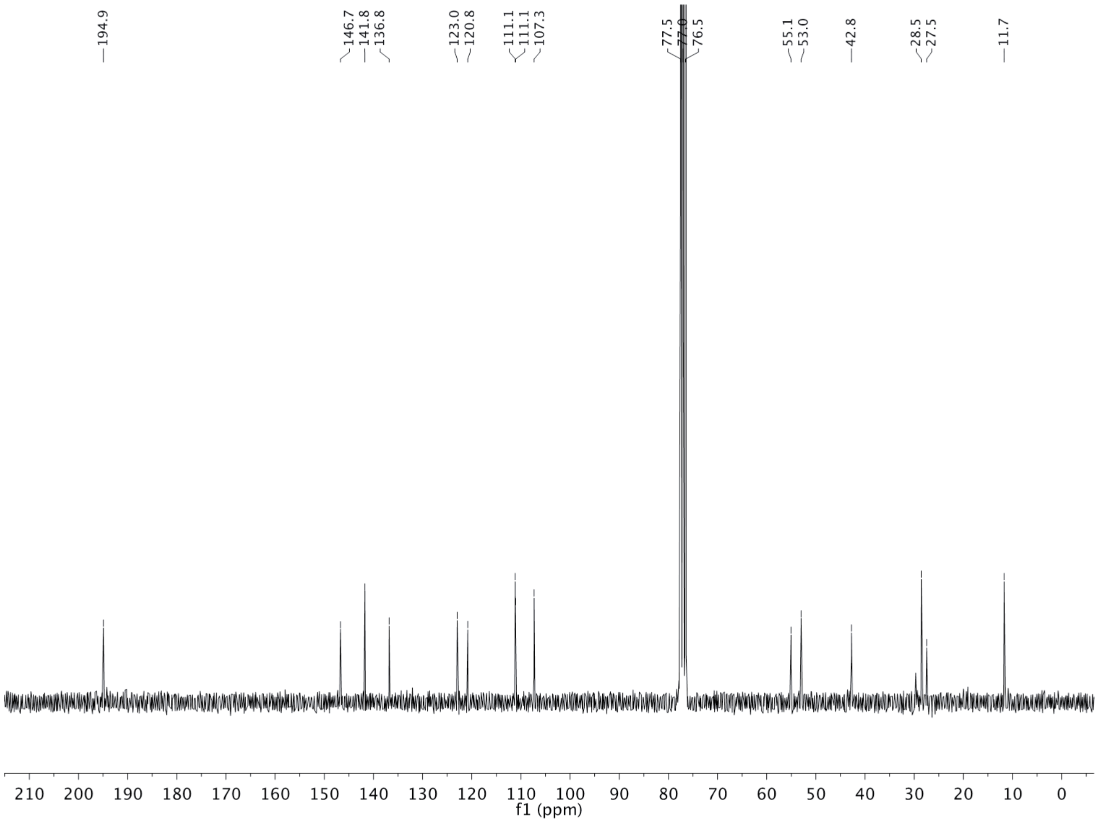

1,1'-[[(1,1,3,3-Tetramethyldisiloxane-1,3-diyl)bis(propane-3,1-diyl)]bis(2-methyl-5-phenyl-1*H*-pyrrole-1,3-diyl)]bis(ethan-1-one) (1k)

^{1}H NMR (250 MHz, $CDCl_3$)

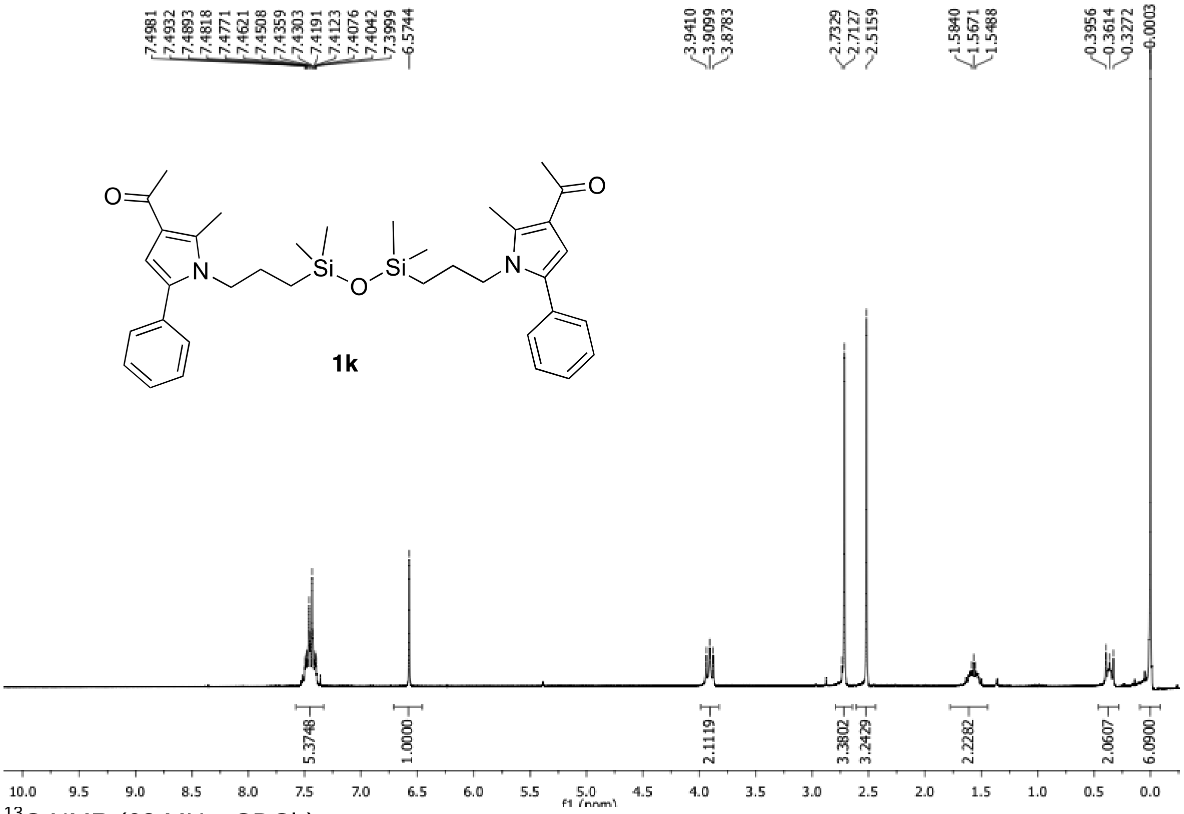

1k

^{13}C NMR (63 MHz, $CDCl_3$)

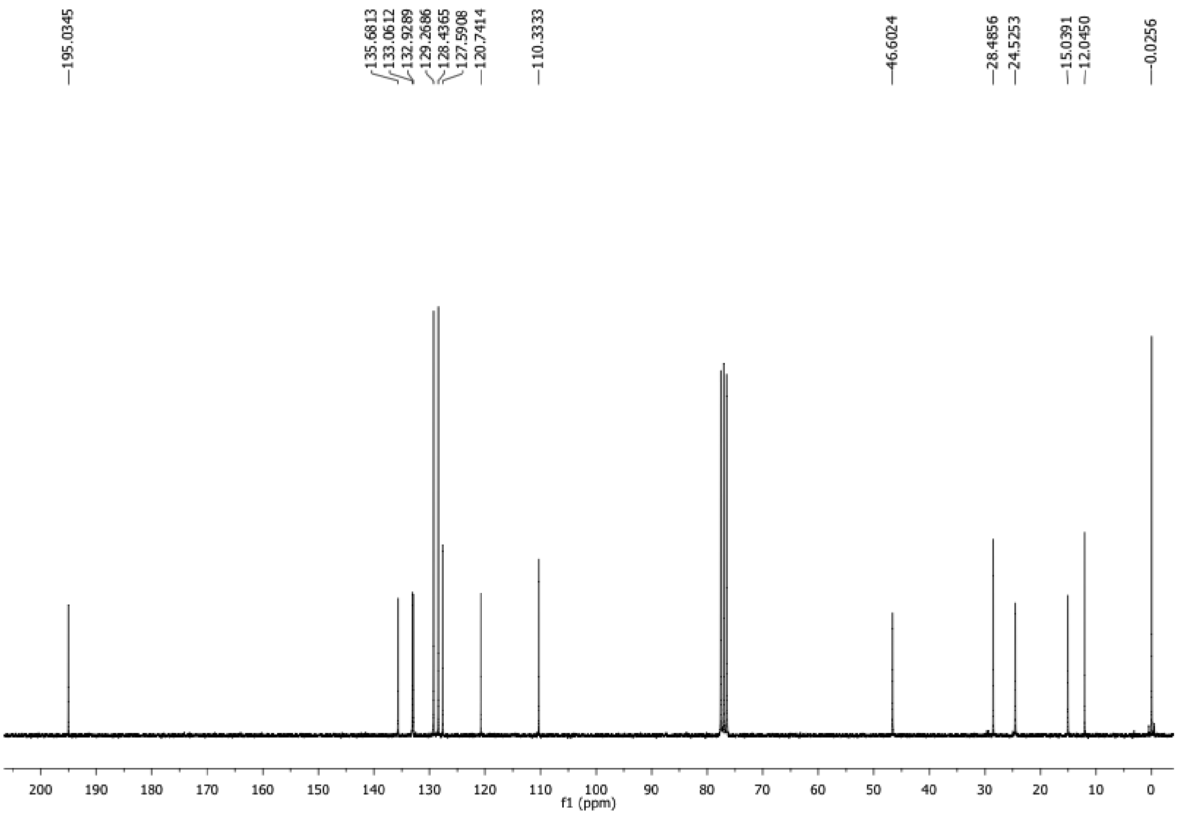

1,1'-[[(1,1,3,3-Tetramethyldisiloxane-1,3-diyl)bis(propane-3,1-diyl)]bis(2-methyl-5-(thiophen-2-yl)-1*H*-pyrrole-1,3-diyl)]bis(ethan-1-one) (1l)

^{1}H NMR (250 MHz, $CDCl_3$)

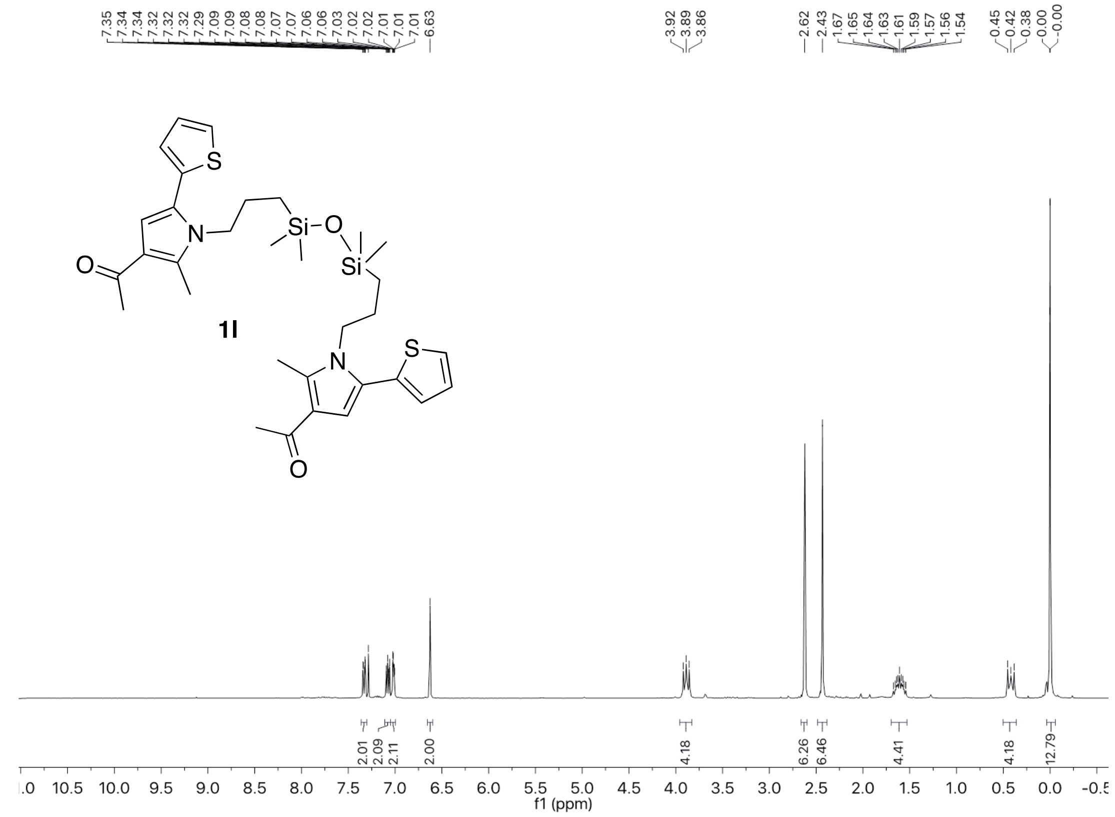

^{13}C NMR (63 MHz, $CDCl_3$)

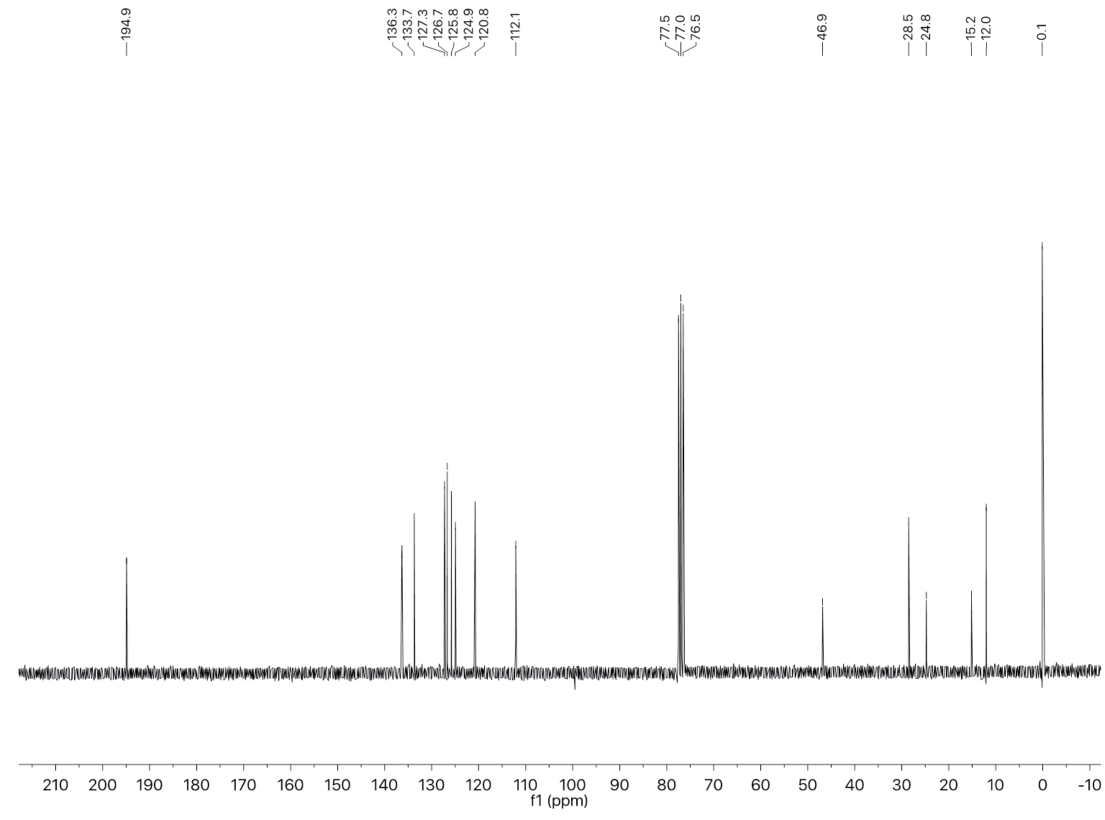

Dimethyl 1,1'-[(1,1,3,3-tetramethyldisiloxane-1,3-diyl)bis(propane-3,1-diyl)]bis[5-(furan-2-yl)-2-methyl-1*H*-pyrrole-3-carboxylate] (1m)

^{1}H NMR (250 MHz, $CDCl_3$)

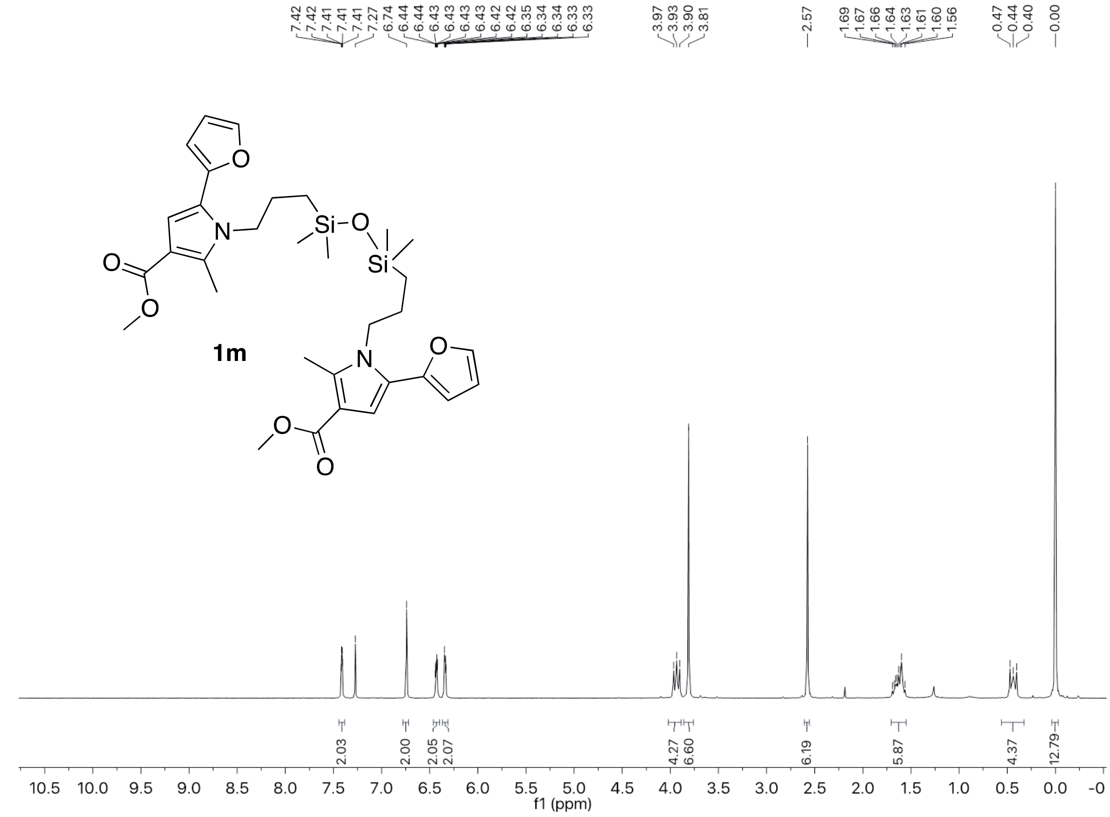

^{13}C NMR (63 MHz, $CDCl_3$)

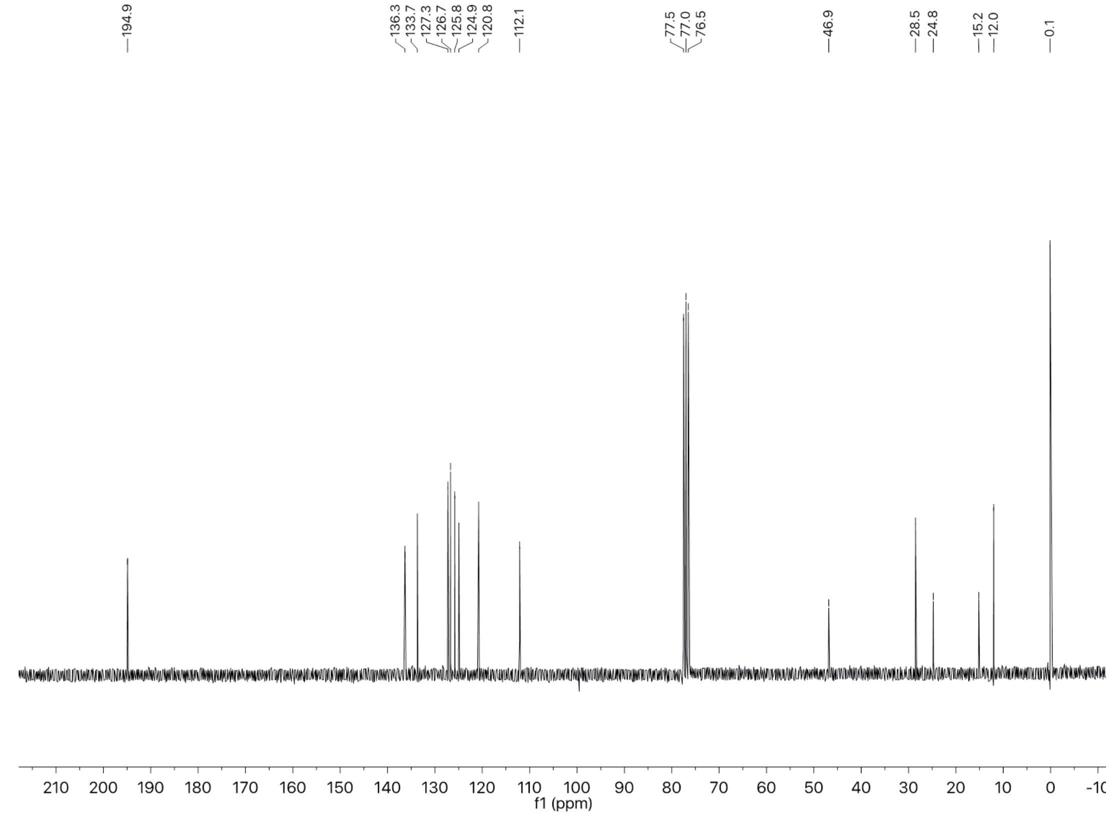

Diethyl 1,1'-[(1,1,3,3-tetramethyldisiloxane-1,3-diyl)bis(propane-3,1-diyl)]bis[2-(2-ethoxy-2-oxoethyl)-5-phenyl-1*H*-pyrrole-3-carboxylate] (1n)

^{1}H NMR (250 MHz, $CDCl_3$)

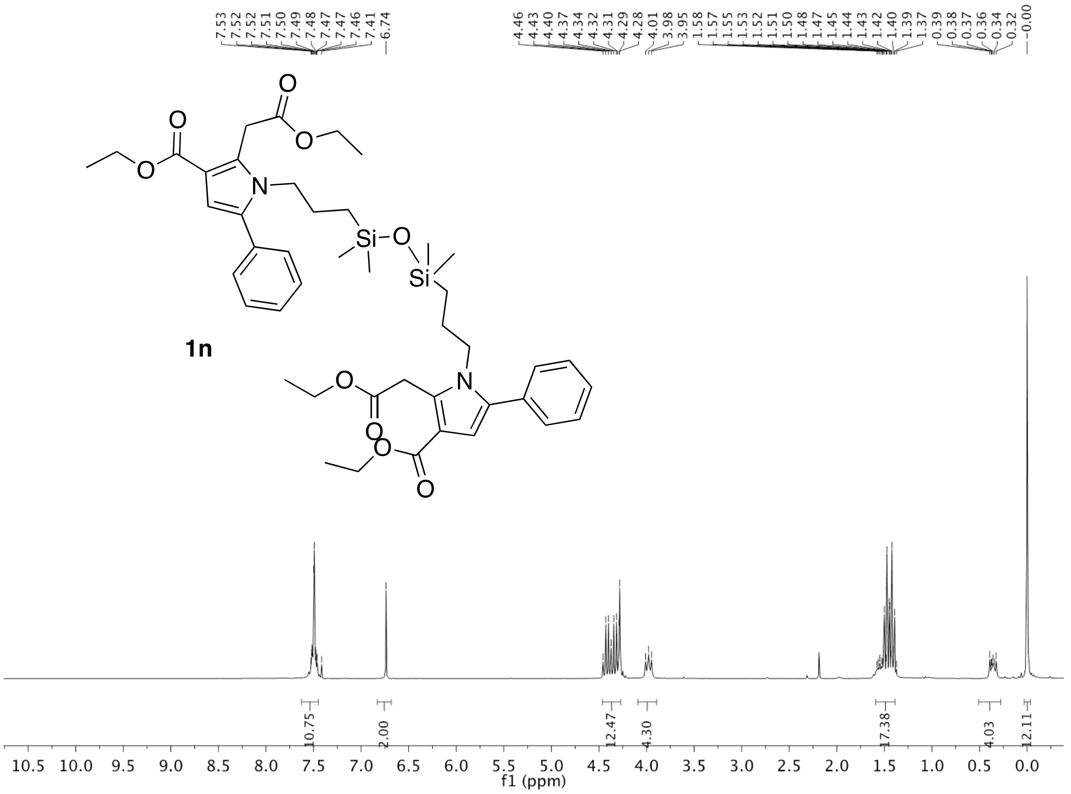

^{13}C NMR (63 MHz, $CDCl_3$)

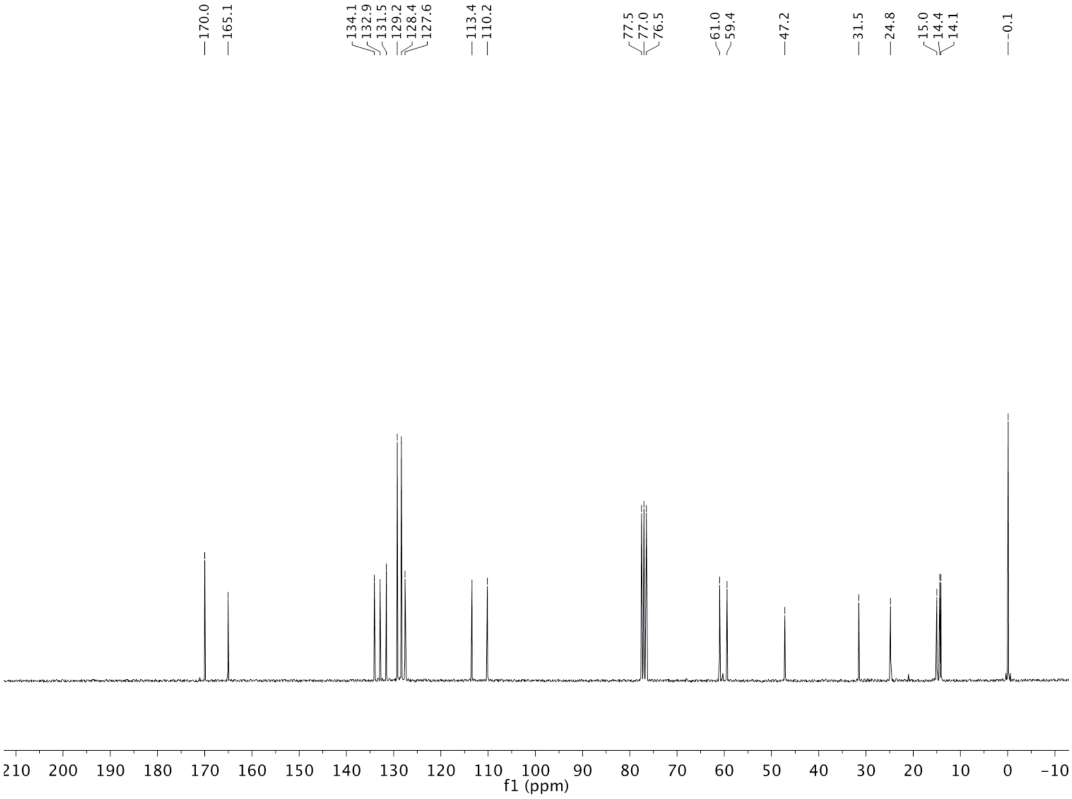

Dimethyl 1,1'-[(1,1,3,3-tetramethyldisiloxane-1,3-diyl)bis(propane-3,1-diyl)]bis(2-methyl-1,4-dihydroindeno[1,2-*b*]pyrrole-3-carboxylate) (1o)

^{1}H NMR (250 MHz, $CDCl_3$)

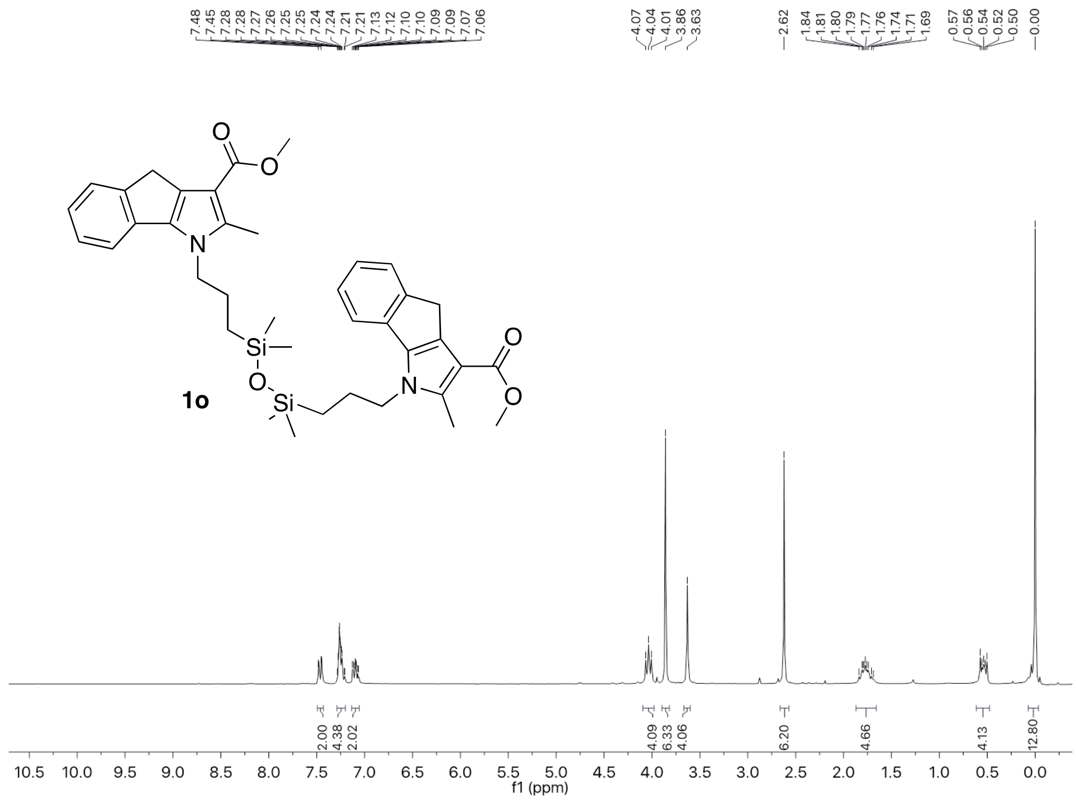

^{13}C NMR (63 MHz, $CDCl_3$)

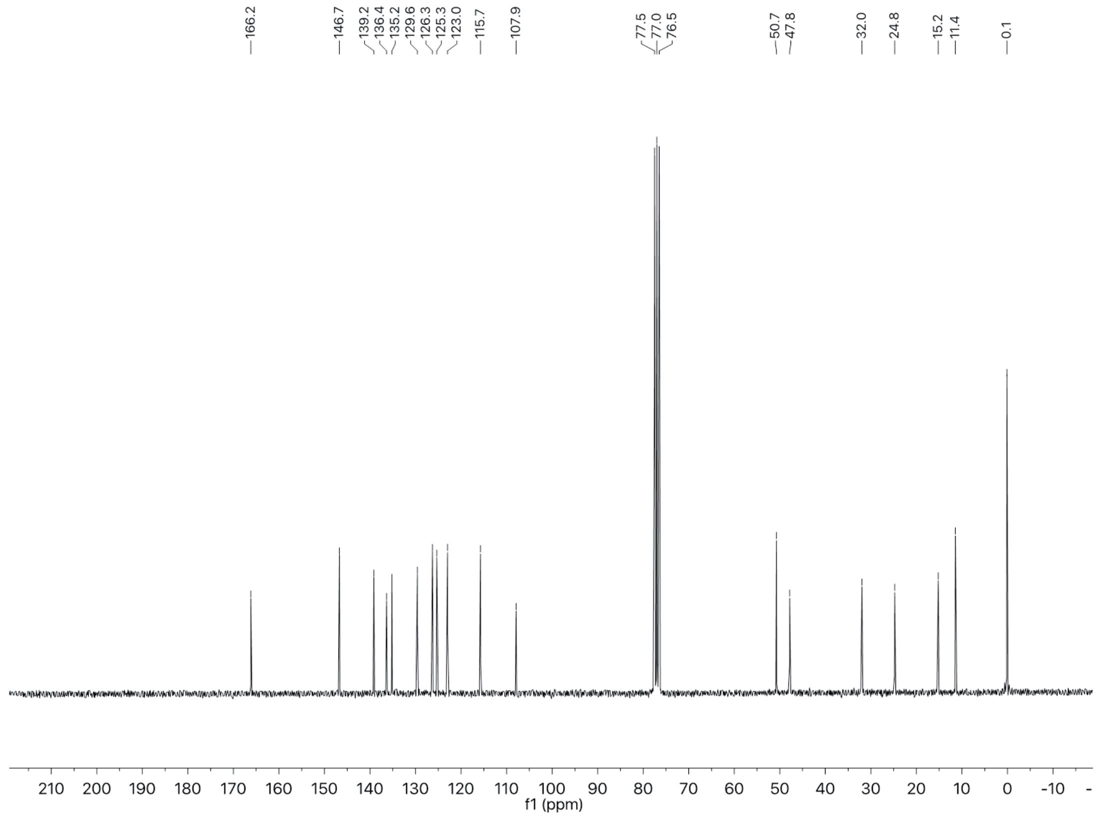

Representative bis-β-enaminone intermediate: Dimethyl 3,3'-(butane-1,4-diylbis(azanediyl)) (2*Z*,2'*Z*)bis(but-2-enoate)

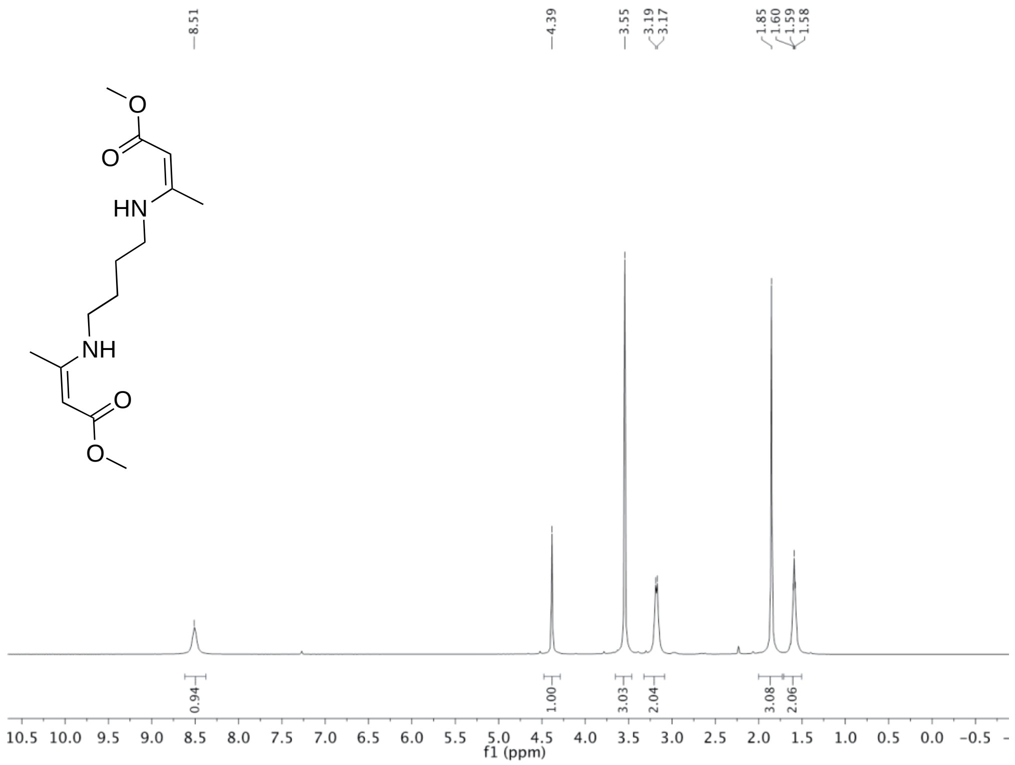

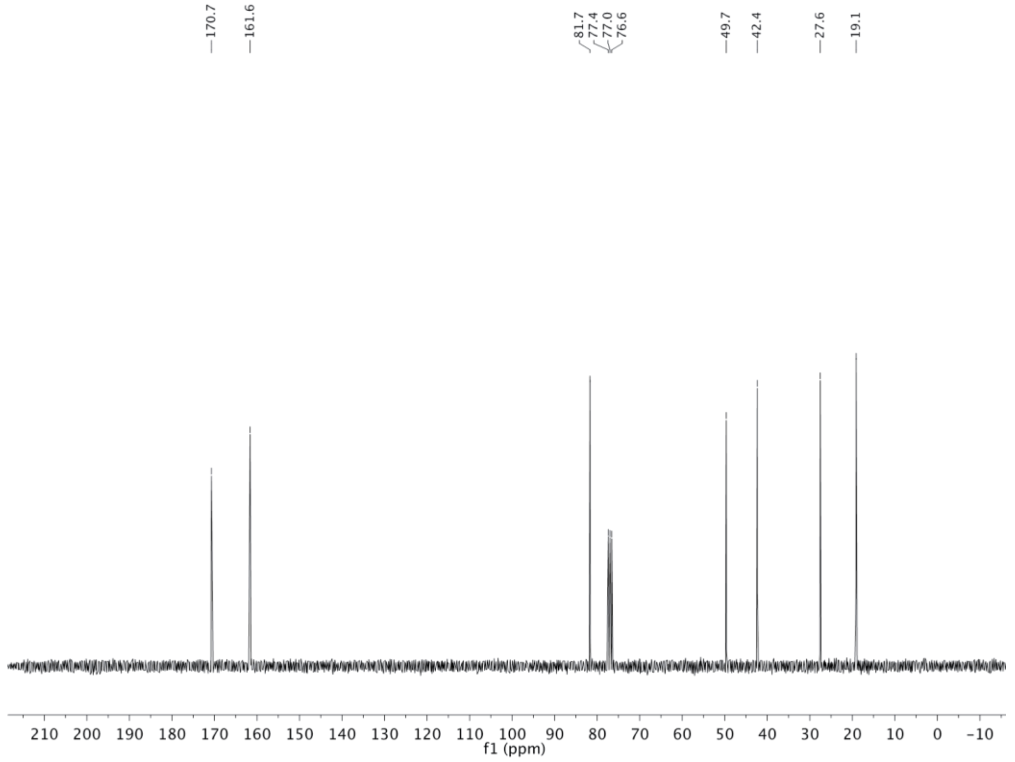

_N_,_N_,_N_-Tris[2-(3-acetyl-5-phenyl-2-methylpyrrol-1-yl)ethyl]amine (8)

^{1}H NMR (250 MHz, $CDCl_3$)

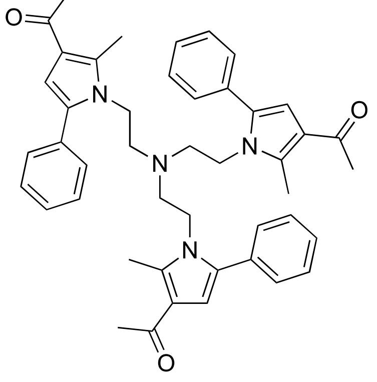

8

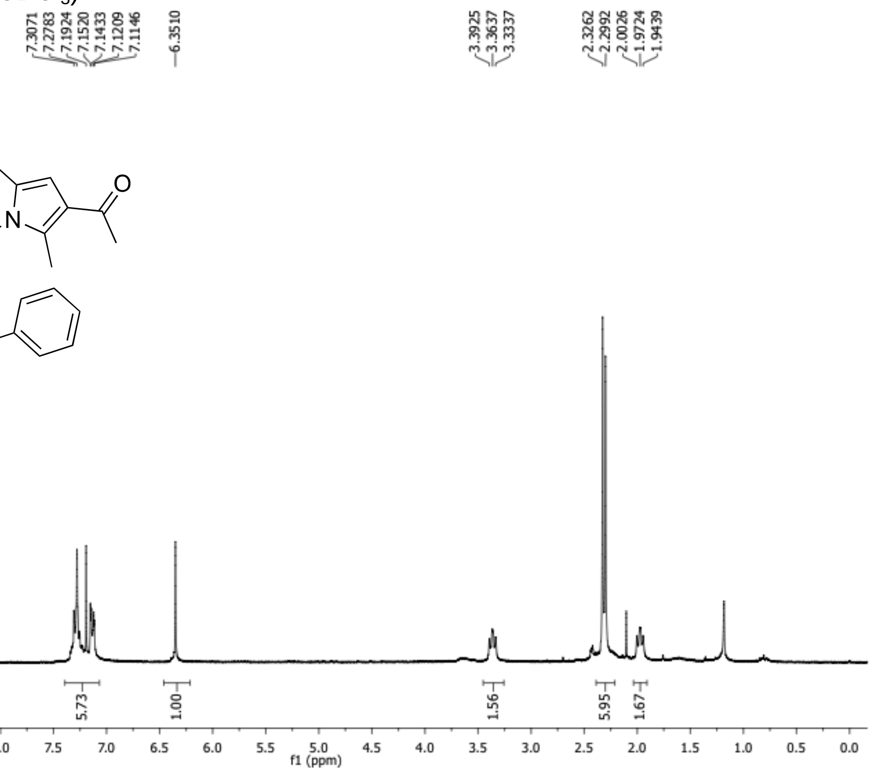

^{13}C NMR (63 MHz, $CDCl_3$)

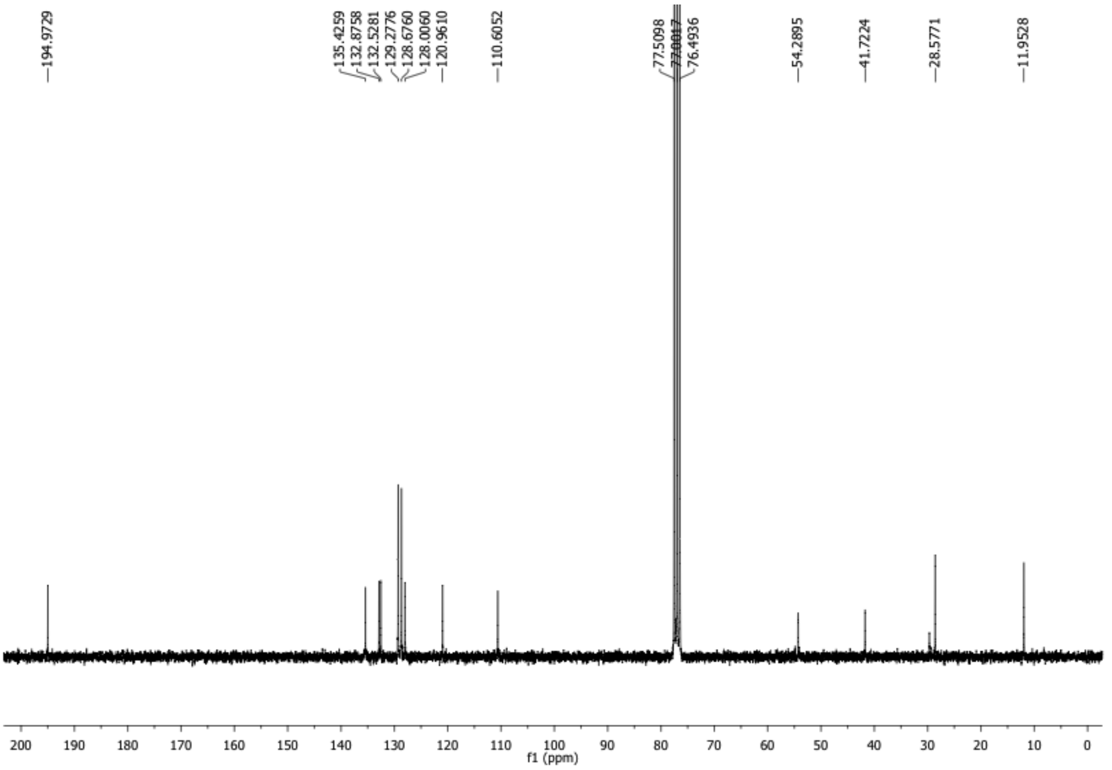

Methyl 1-allyl-5-(furan-2-yl)-2-methyl-1*H*-pyrrole-3-carboxylate (11a)

^{1}H NMR (250 MHz, $CDCl_3$)

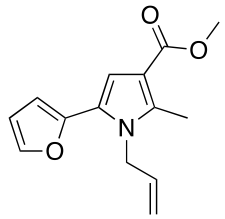

11a

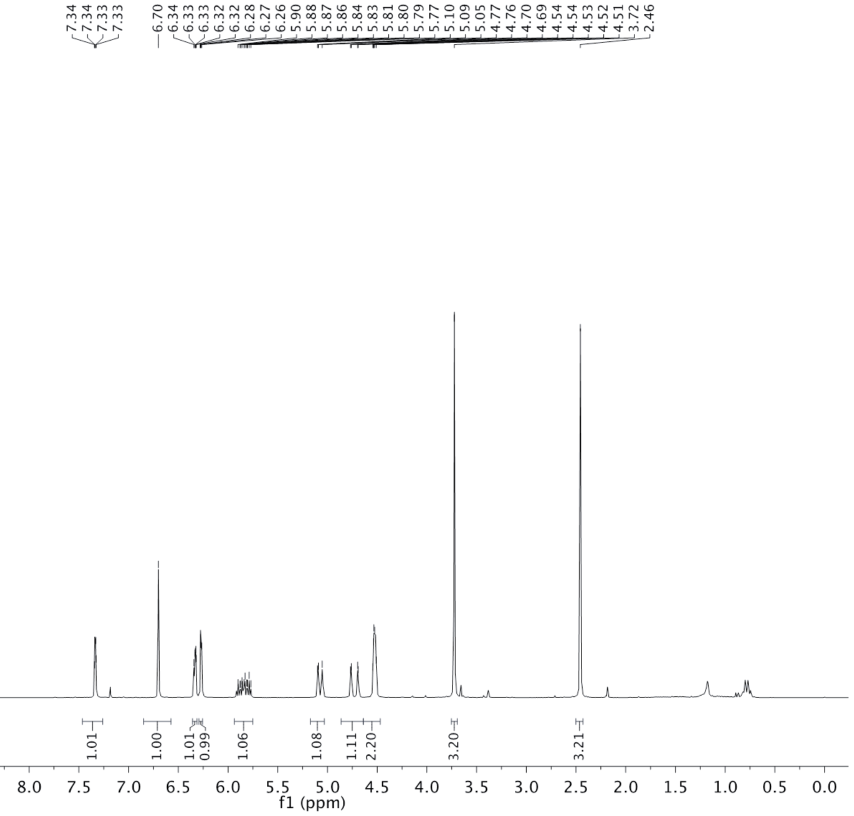

^{13}C NMR (63 MHz, $CDCl_3$)

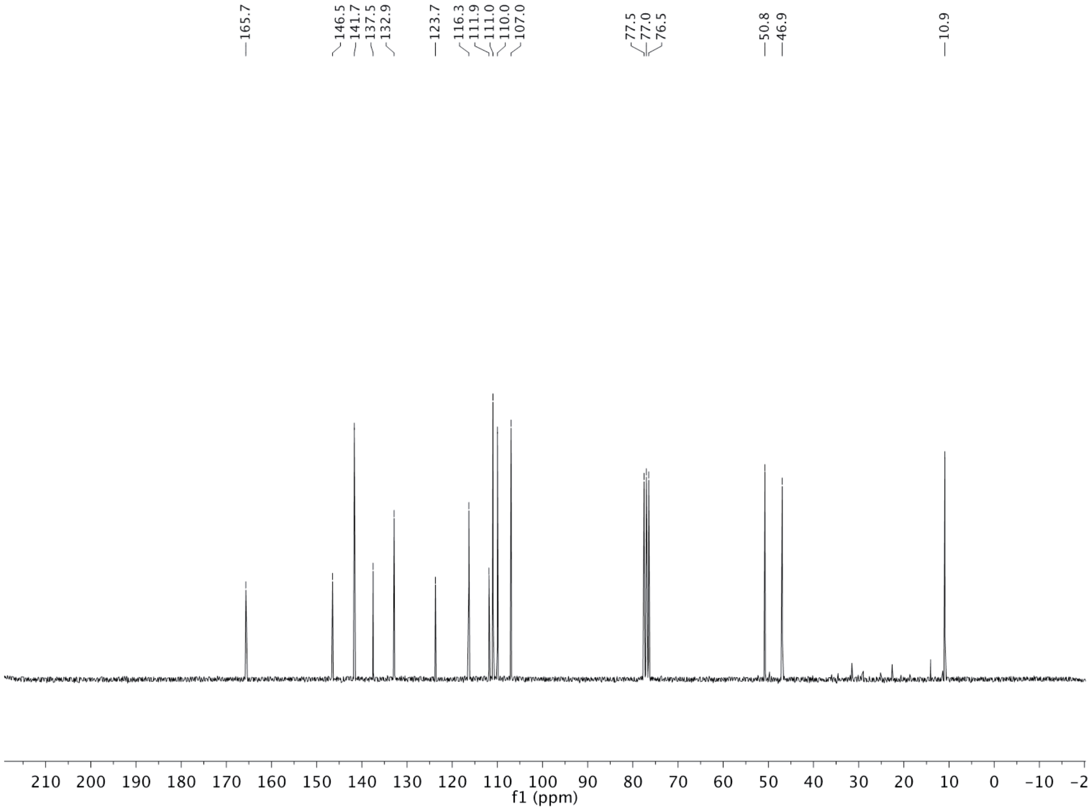

1-(1-Allyl-5-(*tert*-butyl)-2-methyl-1*H*-pyrrol-3-yl)ethan-1-one (11b)

^{1}H NMR (250 MHz, $CDCl_3$)

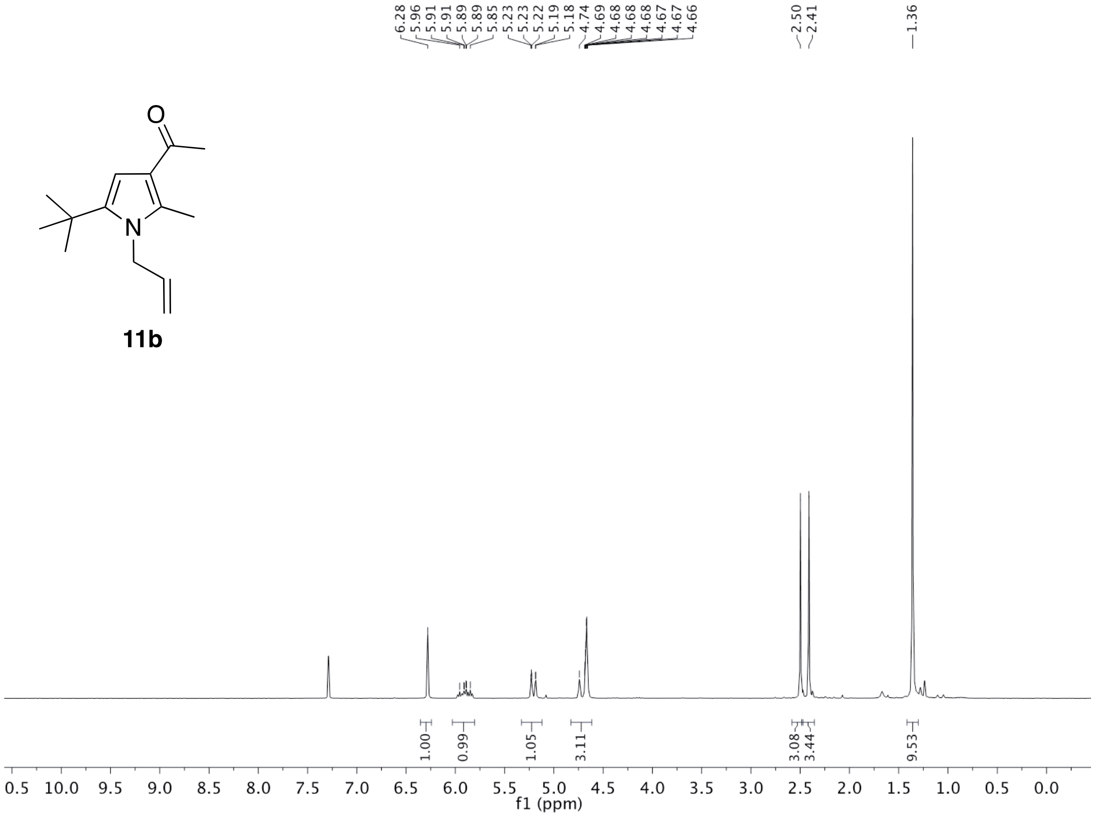

^{13}C NMR (63 MHz, $CDCl_3$)

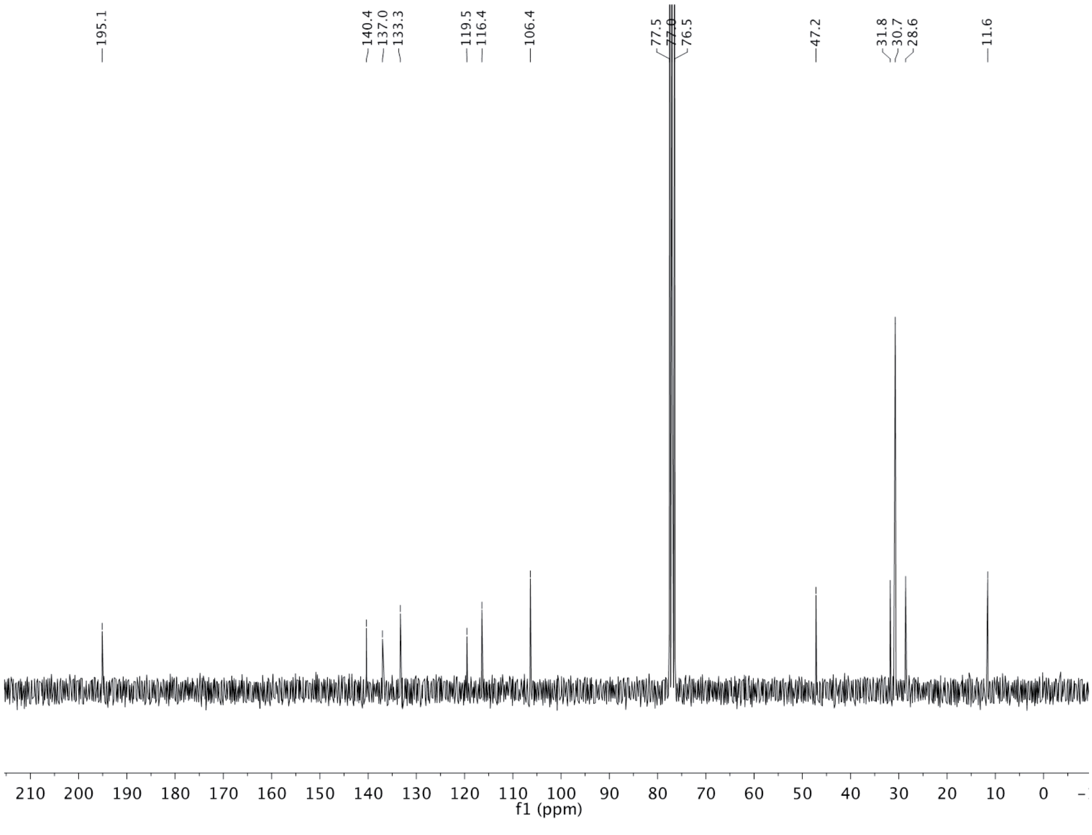

Methyl 1-(but-3-en-1-yl)-2-methyl-5-(thiophen-2-yl)-1*H*-pyrrole-3-carboxylate (11c)
^{1}H NMR (250 MHz, $CDCl_3$)

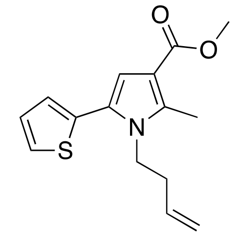

11c

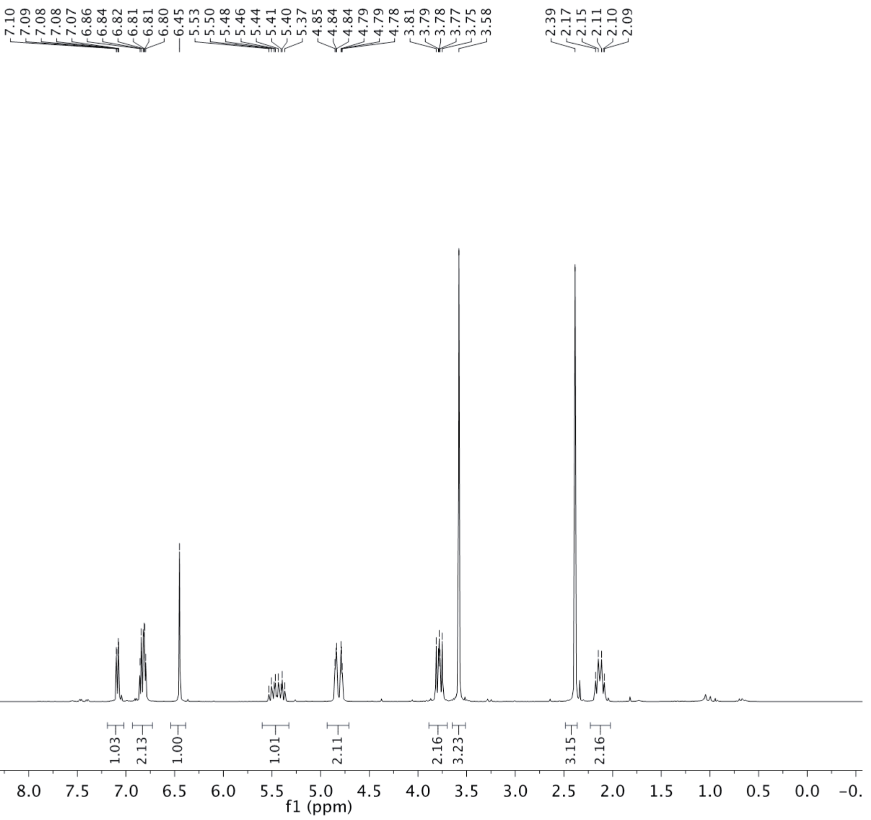

^{13}C NMR (63 MHz, $CDCl_3$)

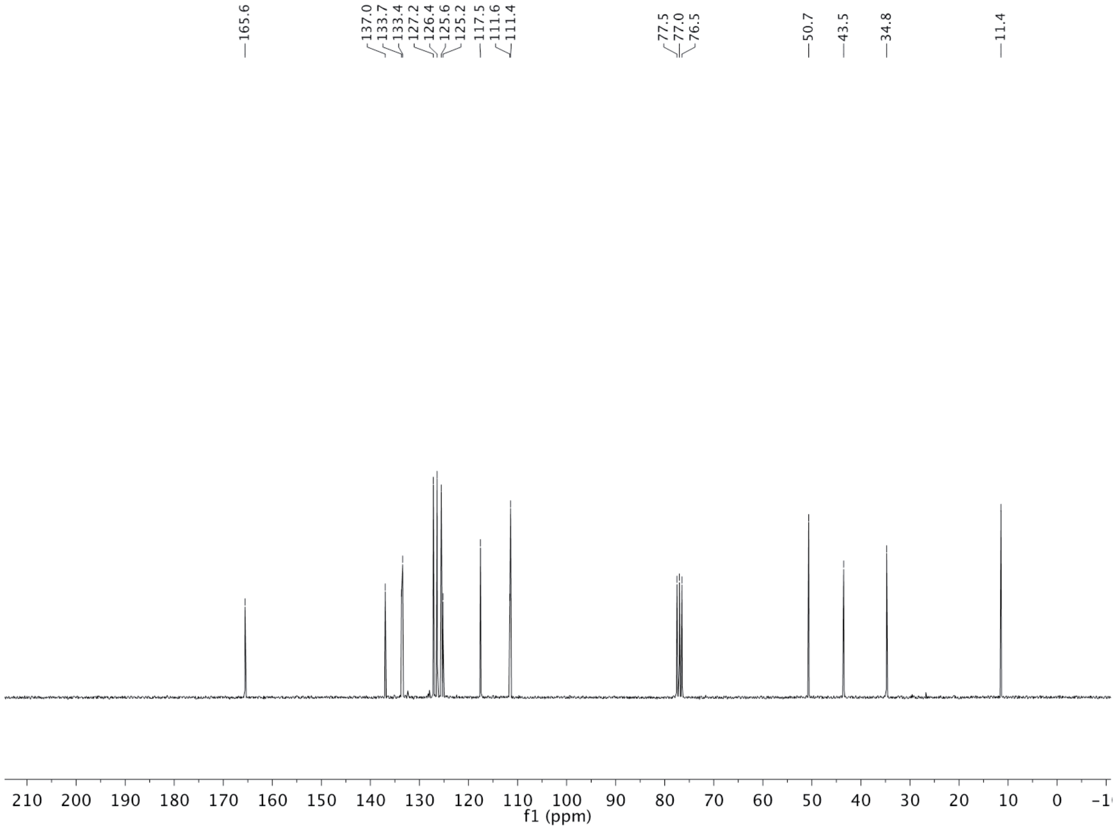

Ethyl 1-allyl-2-(2-ethoxy-2-oxoethyl)-5-(furan-2-yl)-1*H*-pyrrole-3-carboxylate (11d)

^{1}H NMR (250 MHz, $CDCl_3$)

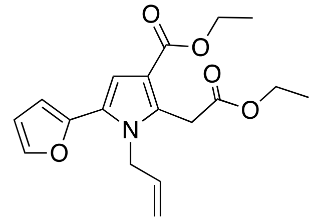

11d

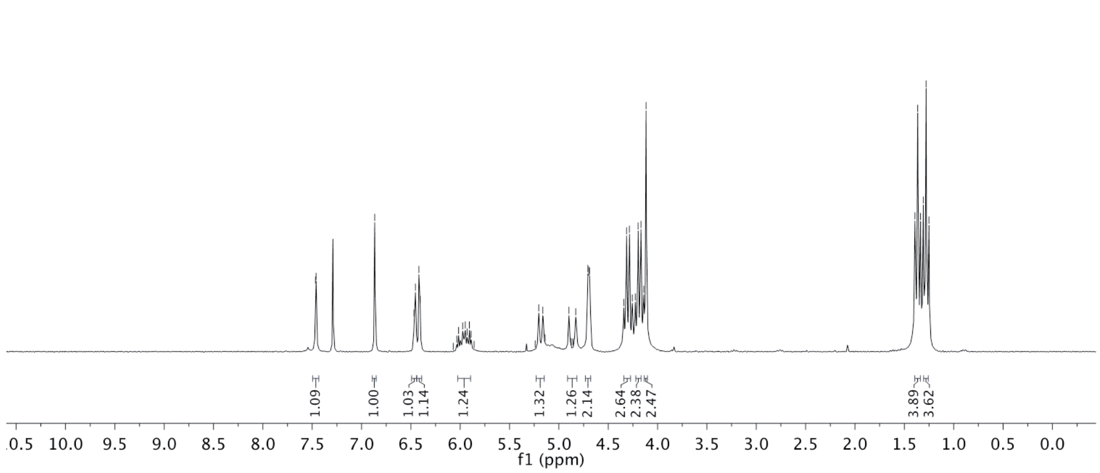

^{13}C NMR (63 MHz, $CDCl_3$)

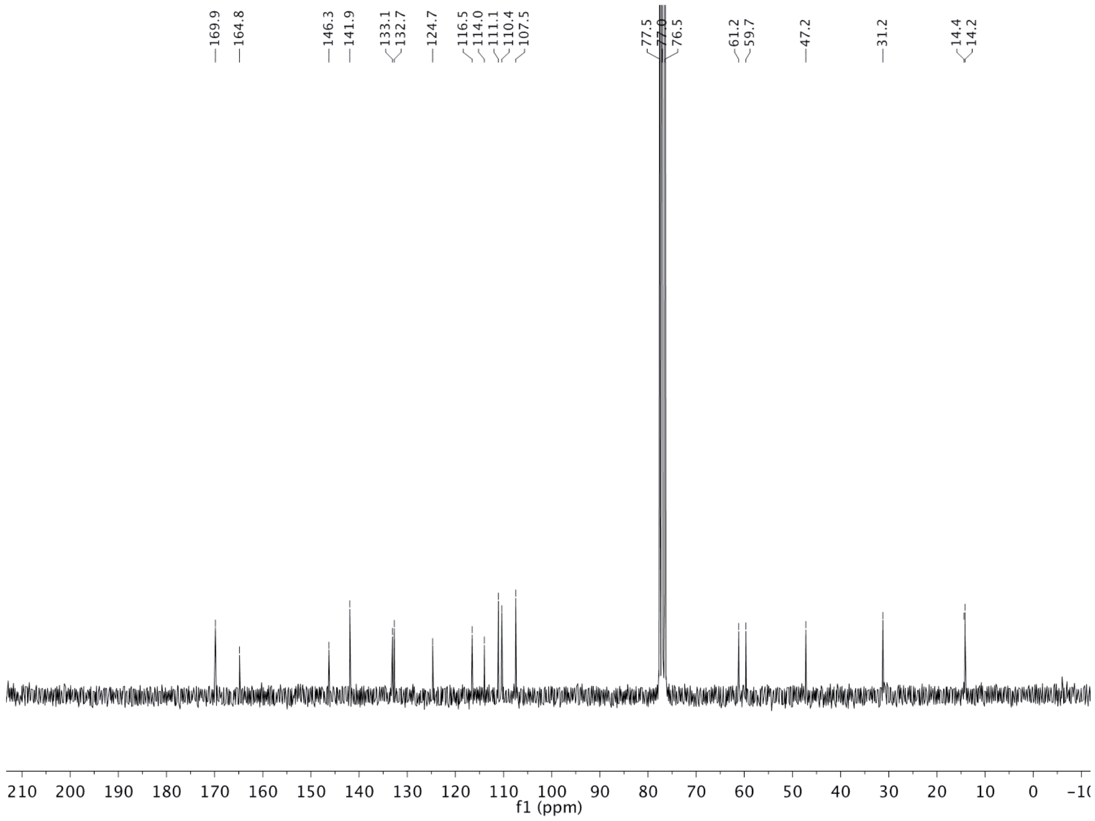

Dimethyl 1,1'-[(*E*) but-2-ene-1,4-diyl]bis[5-(furan-2-yl)-2-methyl-1*H*-pyrrole-3-carboxylate] (12a)

^{1}H NMR (250 MHz, $CDCl_3$)

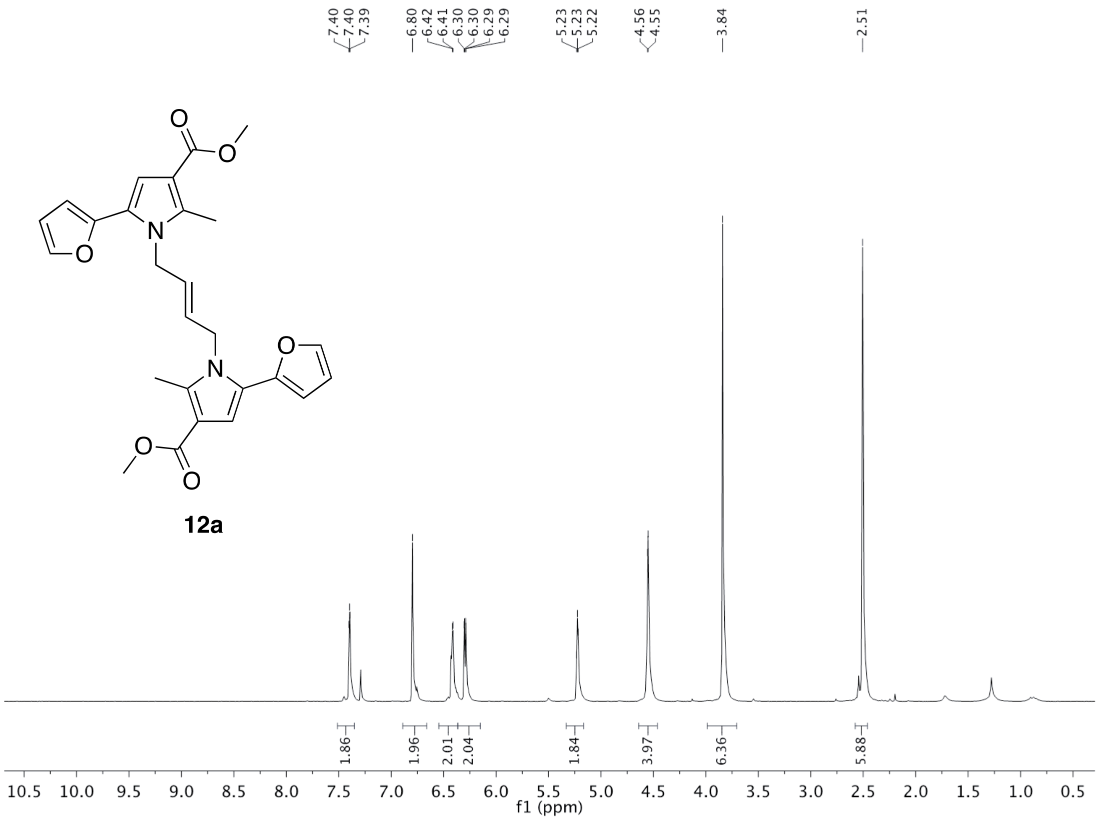

12a

^{13}C NMR (63 MHz, $CDCl_3$)

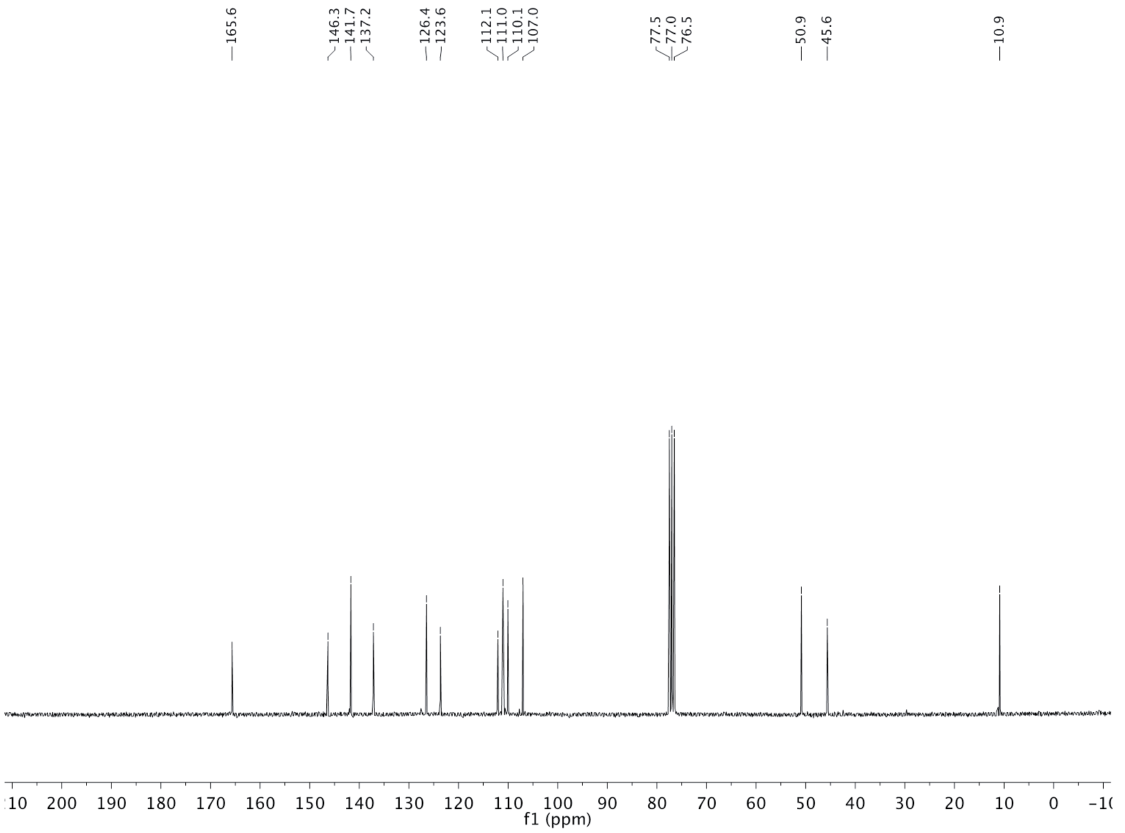

(*E*)-1,1'-(But-2-ene-1,4-diyl)bis[5-(*tert*-butyl)-2-methyl-1*H*-pyrrole-1,3-diyl)]bis(ethan-1-one) (12b)

^{1}H NMR (250 MHz, $CDCl_3$)

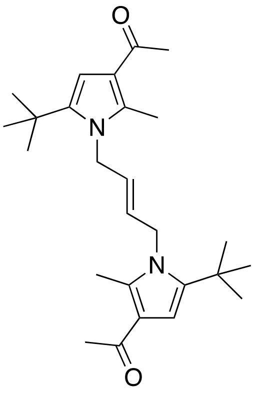

12b

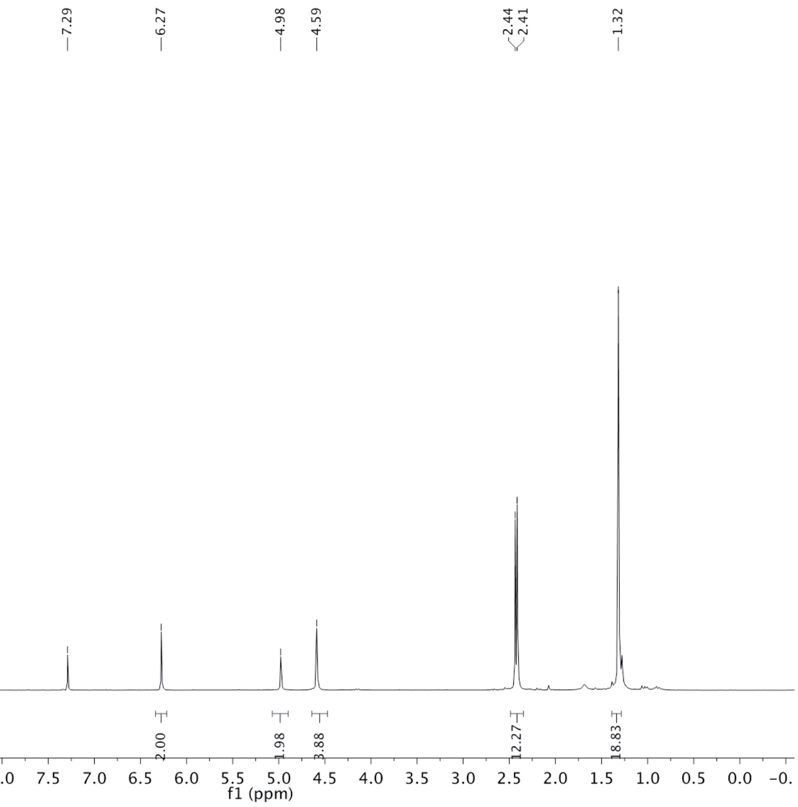

^{13}C NMR (63 MHz, $CDCl_3$)

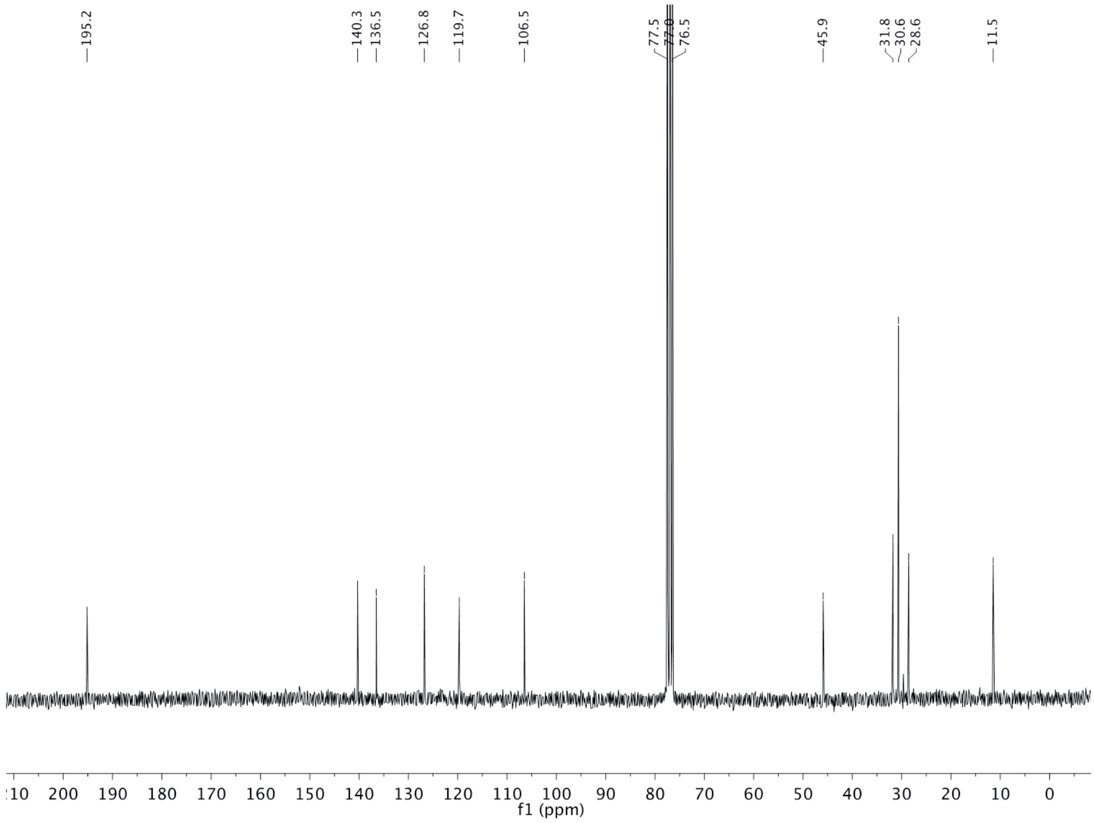

Dimethyl 1,1'-(hex-3-ene-1,6-diyl)bis[2-methyl-5-(thiophen-2-yl)-1*H*-pyrrole-3-carboxylate] (12c)

^{1}H NMR (250 MHz, $CDCl_3$)

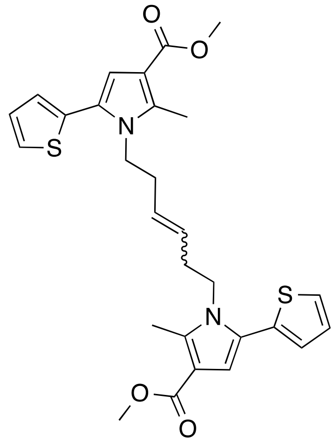

12c

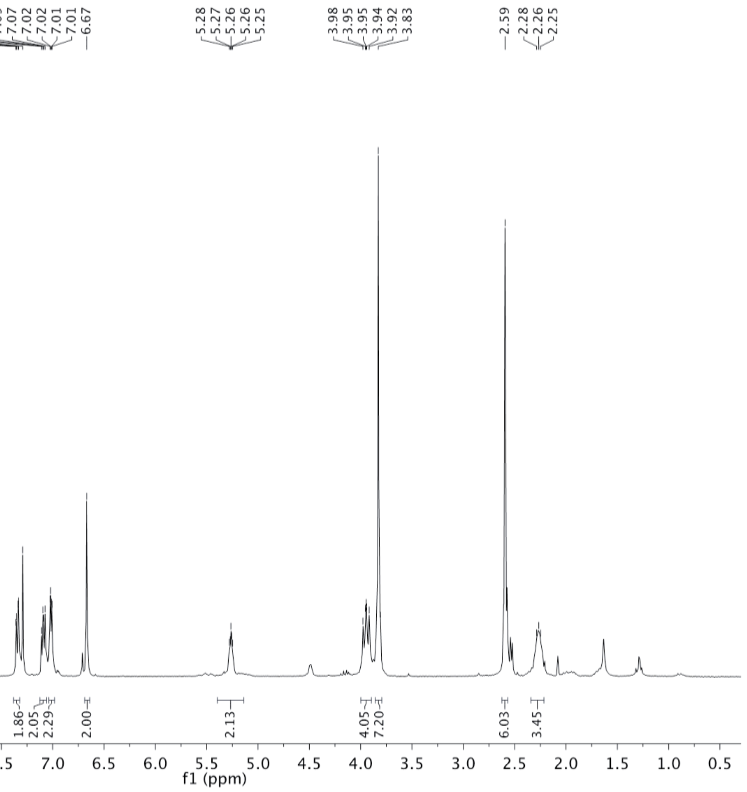

^{13}C NMR (63 MHz, $CDCl_3$)

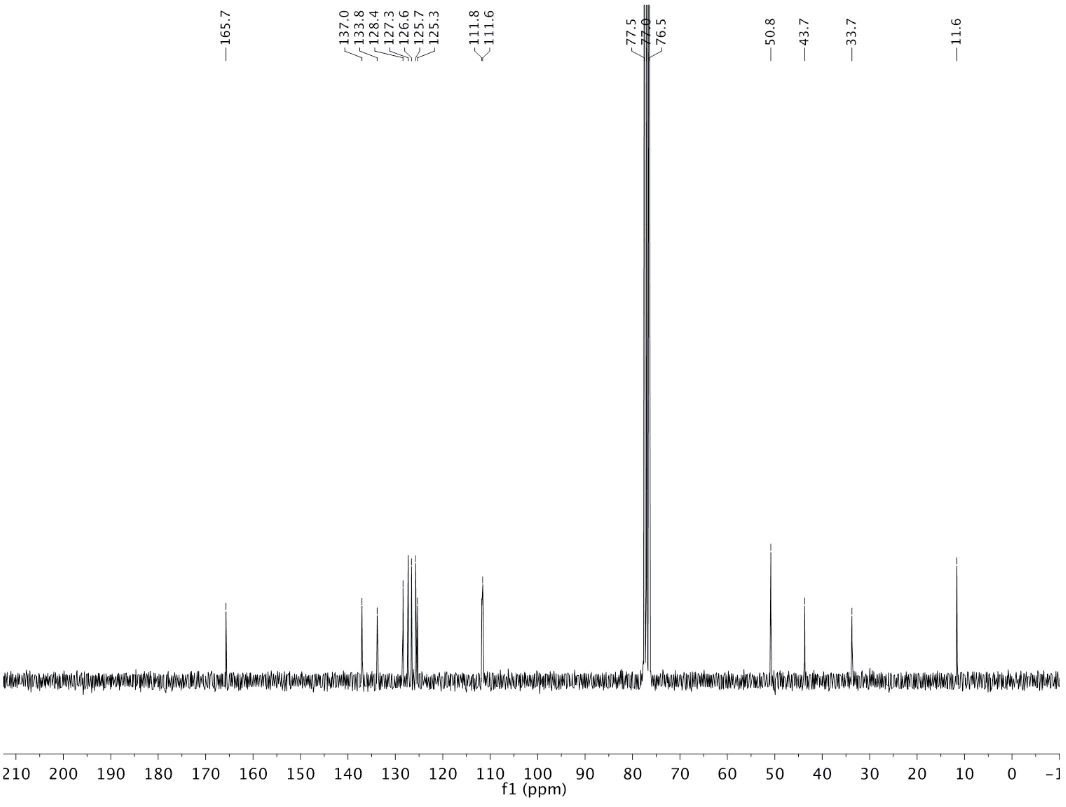

Diethyl 1,1'-[(*E*) (but-2-ene-1,4-diyl)]bis[2-(2-ethoxy-2-oxoethyl)-5-(furan-2-yl)-1*H*-pyrrole-3-carboxylate] (12d)

^{1}H NMR (250 MHz, $CDCl_3$)

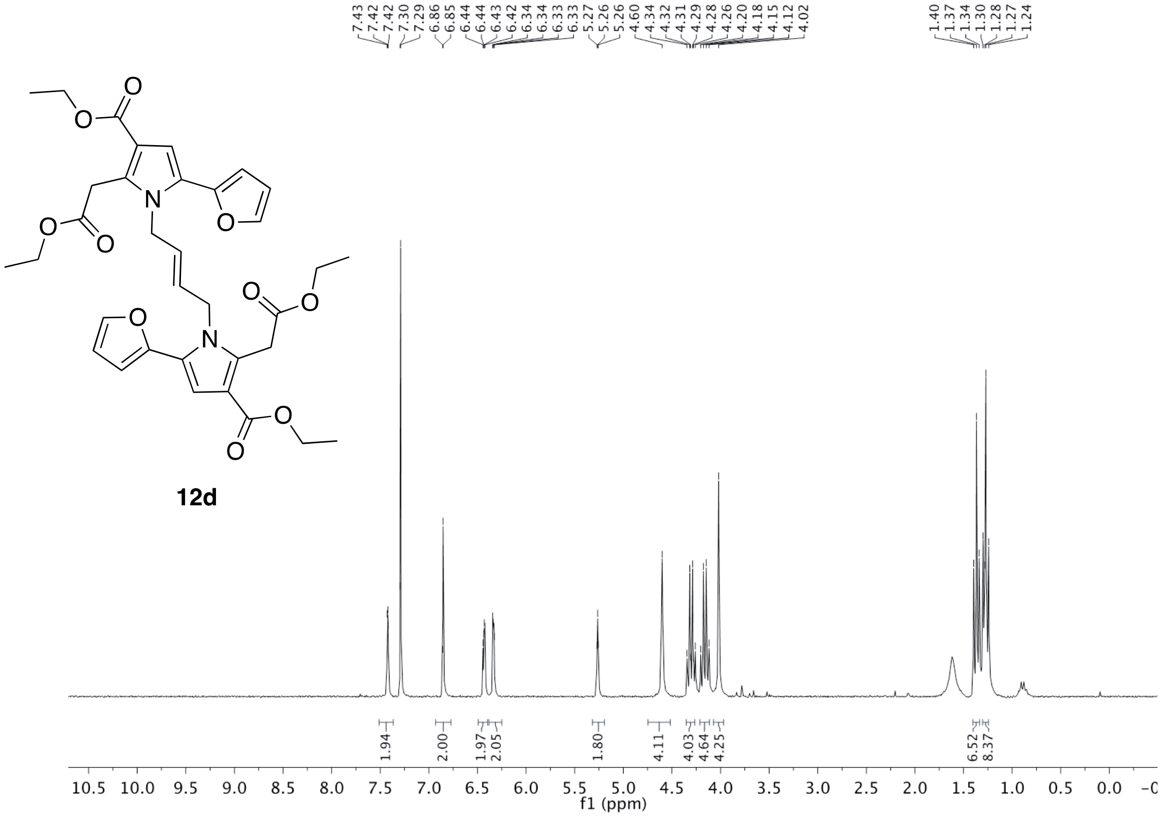

^{13}C NMR (63 MHz, $CDCl_3$)

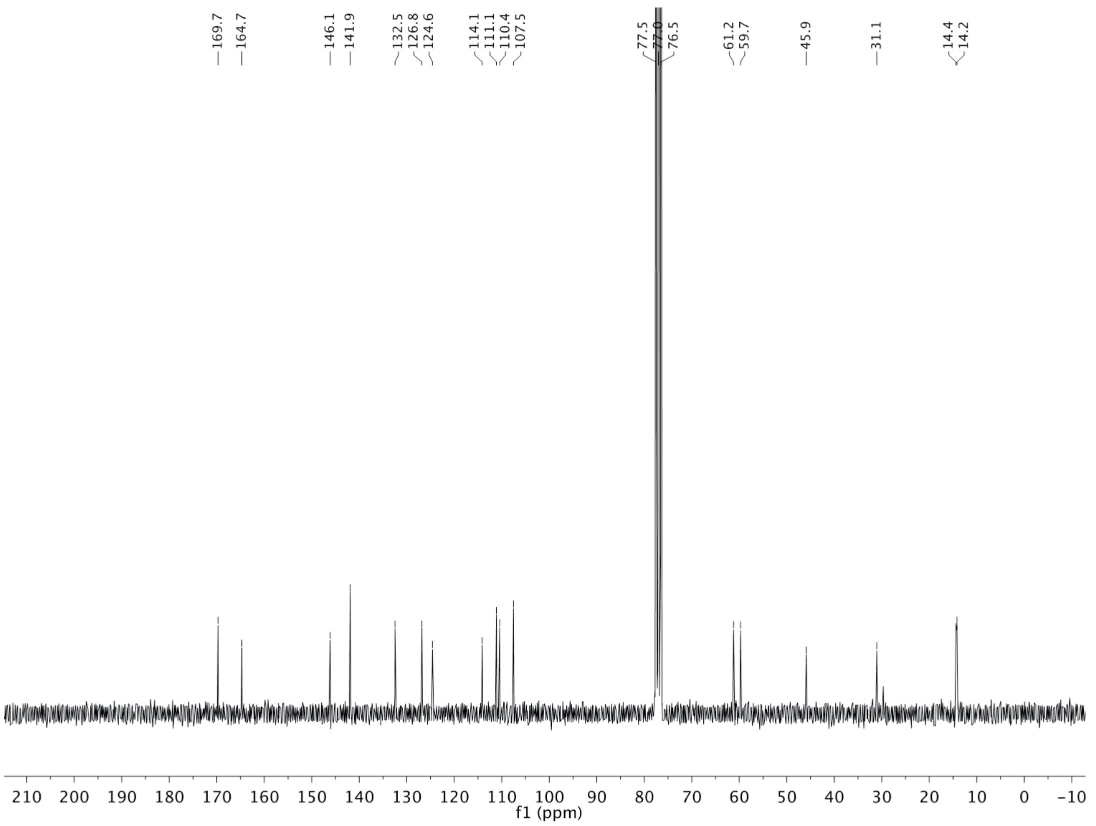

Supplement: File 1 — Experimental details and NMR spectra. [file Beilstein_J_Org_Chem-13-1957-s001.pdf]
